# An 8-Week Self-Administered At-Home Behavioral Skills-Based Virtual Reality Program for Chronic Low Back Pain: Double-Blind, Randomized, Placebo-Controlled Trial Conducted During COVID-19

**DOI:** 10.2196/26292

**Published:** 2021-02-22

**Authors:** Laura M Garcia, Brandon J Birckhead, Parthasarathy Krishnamurthy, Josh Sackman, Ian G Mackey, Robert G Louis, Vafi Salmasi, Todd Maddox, Beth D Darnall

**Affiliations:** 1 AppliedVR, Inc Los Angeles, CA United States; 2 CT Bauer College of Business University of Houston Houston, TX United States; 3 Division of Neurosurgery Pickup Family Neurosciences Institute Hoag Memorial Hospital Newport Beach, CA United States; 4 Department of Anesthesiology, Perioperative and Pain Medicine Stanford University School of Medicine Palo Alto, CA United States

**Keywords:** virtual reality, low back pain, opioids, chronic pain, behavioral health, pain treatment, randomized controlled trial, COVID-19

## Abstract

**Background:**

Chronic low back pain is the most prevalent chronic pain condition worldwide and access to behavioral pain treatment is limited. Virtual reality (VR) is an immersive technology that may provide effective behavioral therapeutics for chronic pain.

**Objective:**

We aimed to conduct a double-blind, parallel-arm, single-cohort, remote, randomized placebo-controlled trial for a self-administered behavioral skills-based VR program in community-based individuals with self-reported chronic low back pain during the COVID-19 pandemic.

**Methods:**

A national online convenience sample of individuals with self-reported nonmalignant low back pain with duration of 6 months or more and with average pain intensity of 4 or more/10 was enrolled and randomized 1:1 to 1 of 2 daily (56-day) VR programs: (1) EaseVRx (immersive pain relief skills VR program); or (2) Sham VR (2D nature content delivered in a VR headset). Objective device use data and self-reported data were collected. The primary outcomes were the between-group effect of EaseVRx versus Sham VR across time points, and the between–within interaction effect representing the change in average pain intensity and pain-related interference with activity, stress, mood, and sleep over time (baseline to end-of-treatment at day 56). Secondary outcomes were global impression of change and change in physical function, sleep disturbance, pain self-efficacy, pain catastrophizing, pain acceptance, pain medication use, and user satisfaction. Analytic methods included intention-to-treat and a mixed-model framework.

**Results:**

The study sample was 179 adults (female: 76.5%, 137/179; Caucasian: 90.5%, 162/179; at least some college education: 91.1%, 163/179; mean age: 51.5 years [SD 13.1]; average pain intensity: 5/10 [SD 1.2]; back pain duration ≥5 years: 67%, 120/179). No group differences were found for any baseline variable or treatment engagement. User satisfaction ratings were higher for EaseVRx versus Sham VR (*P*<.001). For the between-groups factor, EaseVRx was superior to Sham VR for all primary outcomes (highest *P* value=.009), and between-groups Cohen d effect sizes ranged from 0.40 to 0.49, indicating superiority was moderately clinically meaningful. For EaseVRx, large pre–post effect sizes ranged from 1.17 to 1.3 and met moderate to substantial clinical importance for reduced pain intensity and pain-related interference with activity, mood, and stress. Between-group comparisons for Physical Function and Sleep Disturbance showed superiority for the EaseVRx group versus the Sham VR group (*P*=.022 and .013, respectively). Pain catastrophizing, pain self-efficacy, pain acceptance, prescription opioid use (morphine milligram equivalent) did not reach statistical significance for either group. Use of over-the-counter analgesic use was reduced for EaseVRx (*P*<.01) but not for Sham VR.

**Conclusions:**

EaseVRx had high user satisfaction and superior and clinically meaningful symptom reduction for average pain intensity and pain-related interference with activity, mood, and stress compared to sham VR. Additional research is needed to determine durability of treatment effects and to characterize mechanisms of treatment effects. Home-based VR may expand access to effective and on-demand nonpharmacologic treatment for chronic low back pain.

**Trial Registration:**

ClinicalTrials.gov NCT04415177; https://clinicaltrials.gov/ct2/show/NCT04415177

**International Registered Report Identifier (IRRID):**

RR2-10.2196/25291

## Introduction

Chronic low back pain (cLBP) is the most prevalent chronic pain condition worldwide [1]. cLBP can be disabling, costly, and confer suffering to individuals and their families. The incidence and prevalence of cLBP continue to rise despite increasing use of medical treatments such as pharmacology and surgical procedures [2].

An expert evidence review and consensus panel recommended pain education and cognitive behavioral therapy (CBT) as first-line treatments for cLBP [3] with both modalities supplying self-help information for back pain. Beyond the context of back pain, the Centers for Disease Control and Prevention [4] (CDC) and the Centers for Medicare & Medicaid Services (CMS) [5] have recommended nonpharmacologic therapies as first-line treatments for chronic pain, with the latter citing a need to improve patient access to effective treatment options.

CBT for chronic pain engages participants in active pain and symptom self-management [4,6,7]. In group settings, CBT is delivered by trained therapists and typically involves 8-12 two-hour treatment sessions (16-24 hours total treatment time). Manualized session content includes health and pain education; skills training in goal setting, problem solving and action planning; self-regulatory techniques (eg, relaxation, mindfulness); cognitive techniques (eg, thought monitoring and restructuring unhelpful thoughts); and functional goal setting. While CBT has not shown efficacy for reducing pain intensity, it has small to moderate effects for reducing depressive symptoms [7], pain bothersomeness [6,7], and pain catastrophizing [6,7] (Darnall et al, unpublished data) in mixed etiology chronic pain as well as cLBP. Despite demonstrated efficacy for these multisession behavioral pain treatments, access to care remains poor due to barriers such as few trained and available local therapists, health insurance limits, and burdens associated with travel and treatment time [8]. Because of the scope and impact of cLBP, there is an urgent need for effective, accessible, low-risk treatments that are acceptable to people who have back pain. Improved access to behavioral pain care is particularly salient within the context of reduced opioid prescribing for chronic pain nationally [9].

On-demand digital therapeutics may provide home-based access to pain education and skills-based pain self-management. Particularly during the COVID-19 pandemic, home-based behavioral pain care has gained interest, importance, and engagement among patients [10]. Home-based digital pain treatment options include those involving therapist instruction [11,12] (Ziadni et al, unpublished data), as well as fully automated behavioral programs. For the latter, the portfolio of self-treatment options includes computer applications for symptom tracking, education, and treatment [13]; web-based programs that include self-paced multisession skills-based pain management learning modules [14]; and virtual reality (VR) immersive treatment [15].

VR treatment involves using headset devices that fully restrict the vision field to content displayed inside the headset screen; auditory perception is not fully restricted, though the corresponding device-delivered auditory content commands attention. As a treatment modality, VR provides a unique environment comprising 3D visually immersive experiences that are enriched with stereo sounds and elements such as rich colors and scenic environments that enhance elicitation of desired states of arousal and affect. Within the therapeutic context, VR may be flexibly designed and tailored to address the needs of specific conditions (eg, anxiety, depression, pain) [15-19]. The multisensory immersive VR environment stimulates the visual, auditory, and proprioceptive senses, thus engendering the perception that the user is physically located within the virtual environment they are viewing in the headset [20,21]. Mechanistically, the integrated, multisensory, and immersive properties of VR are thought to enhance treatment effects. For example, results from phobia treatment research has suggested noninferiority of VR treatment compared to treatment with a live therapist [18].

In terms of pain, evidence from multiple independent research groups suggests that VR is effective for managing acute pain [22], including pain evoked during medical procedures [23-28], burn wound care [29,30], and in hospitalized patients [31,32]. Researchers of a randomized controlled trial (RCT) conducted in hospitalized patients found that VR yielded the highest efficacy in patients reporting the most severe pain (≥7/10), thereby underscoring its potent analgesic potential [31].

The scientific literature on VR for chronic pain includes studies conducted in complex regional pain syndrome [33], chronic headache/migraine [34], fibromyalgia [35,36], and chronic musculoskeletal pain [37,38]. Two recent reviews and meta-analyses reported VR efficacy for reducing pain and disability (physical rehabilitation) for painful spinal conditions [39] and for orthopedic rehabilitation [40]. Such rehabilitation studies may apply interactive VR in isolation or with kinematic training [41,42]. In addition to small sample sizes, the literature for VR in chronic pain remains limited by studies conducted in experimental or clinical settings (versus home-based and pragmatic studies), a lack of placebo-controlled studies, and studies that have yielded low-quality evidence [39]. Additionally, the literature has been largely restricted to VR content involving distraction or physical rehabilitation and kinematic exercises with little or no content on active behavioral pain management skills acquisition. To address several of these evidence gaps, our group recently conducted an RCT of a 21-day VR program that included chronic pain education and pain relief skills such as diaphragmatic breathing and relaxation response training, and cognition and emotion regulation techniques [15]. Individuals with cLBP or fibromyalgia were randomized to receive either the 21-day VR treatment program or the same treatment content delivered in audio-only format (N=74). At posttreatment, the VR skills-based treatment group evidenced superior reductions in pain intensity and pain-related interference with activity, sleep, mood, and stress compared to the audio treatment group, with results strengthening after 2 weeks. Similar treatment engagement rates between treatment groups supported a conclusion that the immersive effects of VR yielded superior outcomes [15].

This study builds on this prior work and extends it in several ways. First, the VR treatment program being tested (EaseVRx) is 56 days in length, thereby aligning more with traditional and reimbursable behavioral medicine programs such as 8-week chronic pain CBT or mindfulness programs. Second, the VR content was enriched with interoceptive entrainment techniques designed to enhance biofeedback response and learning. Third, the therapeutic VR program includes expanded pain neuroscience education, as well as principles and elements drawn from CBT, mindfulness, and acceptance-based treatments for chronic pain. Fourth, the study includes a VR sham comparator to control for the novelty of the technology and placebo effects. Fifth, data for analgesic medication use were collected.

Our objective was to conduct a placebo-controlled RCT in community-based individuals with cLBP assigned to receive one of two 56-day treatment programs: therapeutic VR (EaseVRx) or Sham VR [43]. We hypothesized that participants assigned to therapeutic VR would evidence superior outcomes for all baseline to posttreatment comparisons compared to participants assigned to Sham VR.

## Methods

### Study Protocol

The study protocol is published elsewhere and provides additional detail [43]. We conducted a single-cohort, double-blinded (participant and analysts), placebo-controlled randomized clinical trial in an online convenience national sample of community-based individuals with self-reported cLBP. Study participants were participating in a longer study of 8.5 months’ duration that involves multiple additional posttreatment assessments not reported here. The study was approved by the Western Institutional Review Board on July 2, 2020, and data collection for this report was completed in November 2020. This report is constrained to the end of treatment time point (day 56).

### Study Sample, Setting, Recruitment, and Participant Compensation

**Figure 1:**
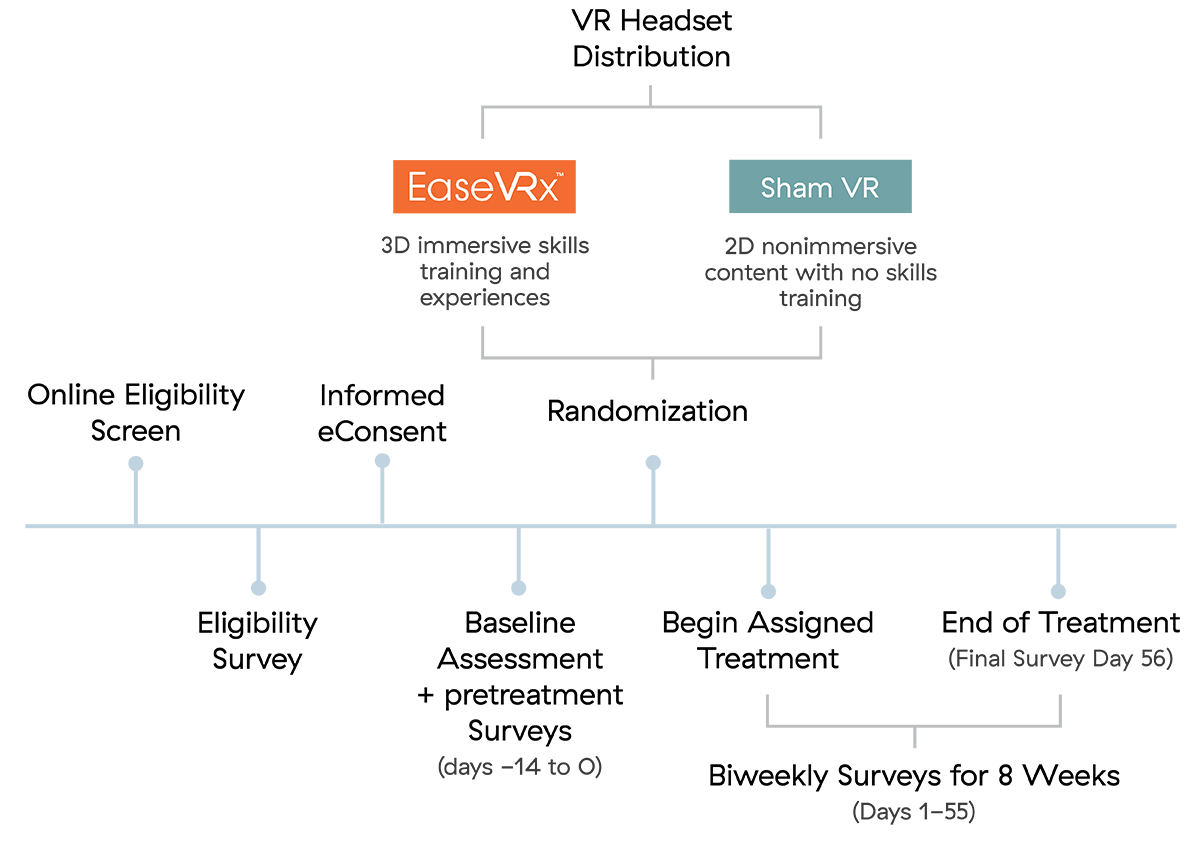
Participant activities.

Community-based individuals with cLBP were recruited nationally through chronic pain organizations (eg, American Chronic Pain Association) and through Facebook online advertisements. Additionally, study advertisements were emailed to professional contacts at several medical clinics with requests to forward among medical colleagues nationally. All study advertisements directed interested individuals with cLBP to the study website for information and invitation to complete an automated online eligibility form that screened for inclusion and exclusion criteria (Textbox 1). This screening process was completed over the phone for 1 individual due to technical difficulties. Individuals determined to be eligible for the study were invited to participate in a study examining the effectiveness of an 8-week VR wellness program in helping them manage chronic lower back pain. If willing to participate, participants completed an electronic informed consent (eConsent; see Multimedia Appendix 1 for the study consent form) and provided their e-signature to complete their enrollment in the study. Figure 1 displays the participant study activities.

Enrolled participants completed a baseline survey and then received 3 pain surveys over a 2-week pretreatment assessment period. Those who completed at least one survey during this pretreatment period progressed to the treatment phase of the study, which included an 8-week VR program (therapeutic VR or Sham VR), twice-weekly surveys during the VR treatment phase, and a final survey administered at treatment completion on day 56. All study procedures occurred remotely.

Study participation was compensated in 2 ways. First, participants received US $6 per completed survey (US $126 possible; prorated) in the form of an Amazon eGift Card after the day 56 survey and upon return of their VR headset (prepaid shipping containers were provided). Second, all participants were eligible to receive a gift VR headset 6 months after their completion of treatment if they completed 16 or more study surveys, confirmed their interest in receiving a VR headset, and returned their VR headset. A total of 77 study participants met these criteria and will receive headsets (Textbox 1).

**Study inclusion and exclusion criteria.**

**Inclusion criteria**

1. Men and women aged 18-85.
2. Self-reported diagnosis of chronic low back pain without radicular symptoms.
3. Chronic low back pain duration of 6 months or more.
4. Average pain intensity of 4 or more out of 10 for the past month.
5. English fluency.
6. Willing to comply with study procedures/restrictions.
7. Access to Wi-Fi.
8. Implicit de facto internet and computer literacy.

**Exclusion criteria**

1. Gross cognitive impairment.
2. Current or prior diagnosis of epilepsy, seizure disorder, dementia, migraines, or other neurological diseases that may prevent the use of virtual reality or adverse effects.
3. Medical condition predisposing to nausea or dizziness.
4. Hypersensitivity to flashing light or motion.
5. No stereoscopic vision or severe hearing impairment.
6. Injury to eyes, face, or neck that impedes comfortable use of virtual reality.
7. Cancer-related pain.
8. Moderate depressive symptoms as indicated by the Patient Health Questionnaire-2 (PHQ-2 [44,45]) depression screen score of 2 or more.
9. Previous use of EaseVRx for pain.
10. Current or recent completion of participation (past 2 months) in any interventional research study.
11. Currently pregnant or planning to become pregnant during the study period.
12. Not expected to have access to Wi-Fi during the study period.
13. Currently works at or has an immediate family member who works for a digital health company or pharmaceutical company that provides treatments for acute or chronic pain.

### Randomization and Blinding

Participants were randomized 1:1 and allocated to 1 of 2 treatment groups: (1) the 56-day pain relief skills VR program (EaseVRx) or (2) a 56-day control VR condition (Sham VR). REDCap Cloud (nPhase, Inc.) was used to apply an automatic and blinded randomization program and ensure equal allocation to both groups. Participants and study statisticians were blinded to treatment group assignment. Statisticians performed blinded analysis of data sets that were randomly labeled group A and group B with statistician unblinding occurring only after posttreatment month 3 data were collected and the data set locked (posttreatment month 3 data not yet analyzed). The 2 study coordinators (LG and IM) unblinded to individual treatment group assignments were not involved in any data analyses. Study participants remain blinded to treatment group assignment until their participation in the larger study is completed (8.5 months after randomization). The larger study quantifies long-term outcomes for the current treatment study; the study protocol and details are published elsewhere [43].

### Study Interventions, VR Headset, and Software

Participants in both treatment groups (EaseVRx and Sham VR) received a Pico G2 4K all-in-one head-mounted VR device at no cost through postal mail. The Pico G2 4K device was used because they are commercially available, widely used, inexpensive, have minimal visual latency, and are easier for participants to use than many other devices. This hardware allows for displaying 3D images (EaseVRx) and 2D images (Sham VR). While each VR device contained software specific to the individual participant’s assigned VR treatment group, all device packaging and directions for use were common to both treatment groups. Participants in this study were provided with online access to instructional materials outlining general use and set up of the headset. Relevant to the EaseVRx group, user exhalation is measured by the microphone embedded in the Pico G2 hardware, offering biodata-enabled immersive therapeutics. Participants in both groups were instructed to complete 1 VR program session daily for 56 days. Study staff monitored participant completion of the twice-weekly surveys and device use. Study staff provided reminders to complete surveys and otherwise were available upon request for technical support. The sections below describe the elements of the study interventions.

### Therapeutic VR (EaseVRx)

Participants randomized and allocated to this treatment group received an immersive multimodal, skills-based, pain self-management VR program, called EaseVRx (AppliedVR), that incorporates evidence-based principles of CBT, mindfulness, and pain neuroscience education. The program content trains users on evidence-based pain and stress management strategies via immersive and enhanced biofeedback experiences. EaseVRx combines biopsychosocial education, diaphragmatic breathing training, relaxation response exercises that activate the parasympathetic nervous system, and executive functioning games to provide a mind–body approach toward living better with chronic pain. The standardized 56-day program delivers a multifaceted combination of pain relief skills training through a prescribed sequence of daily immersive experiences. Each VR experience is 2-16 minutes in length (average of 6 minutes). The VR treatment modules were designed to minimize triggers of emotional distress or cybersickness. Treatment module categories included:

- Pain education: visual and voice-guided lessons establish a medical and scientific rationale for the VR exercises and behavioral medicine skills for pain relief.
- Relaxation/Interoception: scenes that progressively change from busy/active to calm in order to train users to understand the benefits of progressive relaxation.
- Mindful escapes: high-resolution 360 videos with therapeutic voiceovers, music, guided breathing, and sound effects designed to maximize the relaxation response and participant engagement.
- Pain distraction games: interactive games to train the skill of shifting focus away from pain.
- Dynamic breathing: breathing-based biofeedback training in immersive and interactive environments to support self-regulation and relaxation. These modules become increasingly challenging as users increase their skill with diaphragmatic breathing and parasympathetic control.

### Sham VR

**Figure 2:**
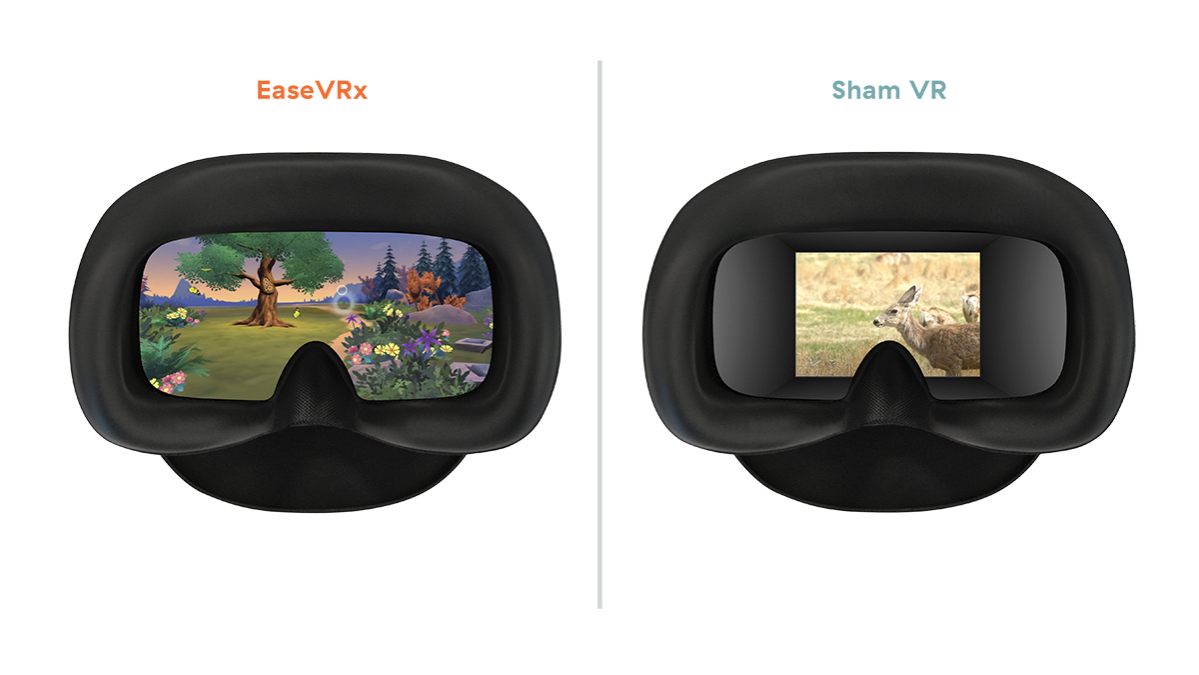
Visual display of EaseVRx (skills-based, interactive, 3D) and Sham VR (non-interactive 2D nature scenes).

In compliance with VR-CORE clinical trial guidelines, we selected an active control that utilizes nonimmersive, 2D content within a VR headset as the most rigorous VR placebo [30]. The Sham VR headset displayed 2D nature footage (eg, wildlife in the savannah) with neutral music that was selected to be neither overly relaxing, aversive, nor distracting. The experience of Sham VR is similar to viewing nature scenes on a large-screen television and is not interactive. Twenty videos were rotated over the 56 sessions, with average duration of sessions closely matching those of EaseVRx (Figure 2).

### Research Standards and Compliance

In accordance with the Initiative on Methods, Measurement, and Pain Assessment in Clinical Trials (IMMPACT) recommendations, we included multiple measures to evaluate the importance of change in outcomes across 4 recommended domains: pain intensity, health-related quality of life and functioning, and ratings of overall improvement [46-48]. Additionally, measures and individual items were included to align directly with the National Institutes of Health (NIH) Pain Consortium’s *Report on Research Standards for Chronic Low Back Pain* [49] or assess the domains recommended in the report. The study was performed in accordance with the CONSORT (Consolidated Standards of Reporting Trials) guidelines [50] (see Multimedia Appendix 2 for the completed checklist) and the recommended extension for reporting of psychological trials [51]. The Western Institutional Review Board approved the study (Puyallup, WA).

### Data Collection and Time Points

Data collection included electronic participant-reported measures and objective VR device use data collected from the VR devices.

Data were collected across 3 phases of the study: pretreatment (days –14 to 0), active treatment (days 1-55), and end of treatment (day 56). The 14-day pretreatment phase involved administering the pain surveys 5 times (baseline, days –10, –7, –3, and 0); these measures were averaged within participants to establish a single pretreatment score for each variable assessed. During the 8-week active treatment phase, surveys were distributed biweekly (15 total surveys during treatment) and at end of treatment on day 56. Accordingly, there were 17 time points per participant.

### Study Variables and Measures

Table 1 outlines the timeline of variable assessment. This section details the measurement and methods used to assess each variable.

**Table 1:** Timeline of variable assessment.

Variables	Pretreatment phase (Days –14 to 0)		Treatment phase (Days 1 to 55)	End-of-treatment (Day 56)
	Baseline	Biweekly surveys	Biweekly surveys	Final assessment
Demographics and pain duration	X			
Pain intensity	X	X	X	X
Pain interference with activity, mood, sleep, stress	X	X	X	X
Patient’s Global Impression of Change				X
Physical function	X			X
Sleep disturbance	X			X
Pain self-efficacy	X			X
Pain catastrophizing	X			X
Chronic pain acceptance	X			X
Prescription opioid use	X			X^a^
Over-the-counter analgesic medication use				X
Motion sickness and nausea				X^a^
Treatment satisfaction				X^a^
Virtual reality device use			X	X
System usability				X

^a^Because of a system error, these data were not captured at Day 56 as intended but at 1-month posttreatment.

#### Demographics and Pain Duration

Demographic variables included age, gender, level of education, race, ethnicity, employment status, annual household income, relationship status, duration of back pain (years since onset), state of residence, and zip code. To perform geospatial coding, rural–urban commuting area (RUCA) codes were downloaded from a public data set provided by the United States Department of Agriculture Economic Research Service [52]. Participants were classified as rural or urban based on their zip code. Finally, duration of time since pain onset was assessed.

#### Average Pain Intensity

The Defense and Veterans Pain Rating Scale (DVPRS) [53] was used to measure average pain intensity over the previous 24 hours using an 11-point numeric rating scale (0=no pain; 10=as bad as it could be and nothing else matters). Average pain intensity was assessed at baseline, pretreatment, during treatment, and at end of treatment on day 56.

#### Pain Interference With Activity, Mood, Sleep, and Stress

The DVPRS interference scale (DVPRS-II) was used to measure pain interference with activity, sleep, mood, and stress over the past 24 hours [54] (0=does not interfere; 10=completely interferes). Pain interference was assessed at baseline, pretreatment, during treatment, and at end of treatment on day 56.

#### Patient’s Global Impression of Change

Aligning with IMMPACT recommendations for pain research [47], Patient’s Global Impression of Change (PGIC) was assessed on day 56 (end of treatment) using the question, “Since the beginning of VR treatment, how would you describe the changes (if any) in activity limitations, symptoms, emotions and overall quality of life related to your low back pain?” on a 7-point scale ranging from 1 (No change or condition is worse) to 7 (A great deal better, and a considerable improvement that has made all the difference).

#### Physical Function and Sleep Disturbance (PROMIS)

The NIH Physical Function and Sleep Disturbance (PROMIS) [55] short-form measures were used to assess physical function (version 6b) [56] and sleep disturbance (version 6a) [57] over the past 7 days. Higher scores on physical function signify greater function whereas higher scores for sleep disturbance reflect greater symptom severity. The conversion table within the scoring manuals, made available from the Person-Centered Assessment Resource [58,59], was used to calculate the individual short-form T scores using the Item Response Theory scoring algorithms. Specifically, based on published item parameters, T scores (latent trait estimates) are computed for each individual’s response pattern using the Bayesian expected a posteriori method [60-62]. Widely applied in pain research [63-65], these measures were administered at baseline and posttreatment day 56.

#### Pain Catastrophizing

The 13-item Pain Catastrophizing Scale (PCS) [66] is a validated instrument widely used clinically and in pain research to assess patterns of negative cognition and emotion in the context of actual or anticipated pain. Despite having discrete subscales for rumination, magnification, and feelings of helplessness related to pain, prior work has shown that the PCS operates unidimensionally [67] (Cook et al, unpublished data). Aligning with prior work [15] and the goal of brevity, the following 4 PCS items were used: “It’s terrible and I think it’s never going to get any better,” “I become afraid that the pain will get worse,” “I can’t seem to keep it out of my mind,” and “I keep thinking about how badly I want the pain to stop.” Respondents rate the frequency with which they experience such thoughts on a scale from 0 (Not at all) to 4 (All the time). Scores for the 4 items were summed to create a total score and index for pain catastrophizing. This measure was administered at baseline and on day 56.

#### Pain Self-Efficacy

Pain self-efficacy was assessed generally and also within the context of VR. For general pain self-efficacy, the 2-item Pain Self-Efficacy Questionnaire (PSEQ-2) is a validated instrument used to assess respondents’ confidence in their ability to engage in various daily activities despite their chronic pain [68]. The PSEQ-2 comprises the following 2 items: “I can still accomplish most of my goals in life, despite the pain,” and “I can live a normal lifestyle, despite the pain.” Respondents use a 5-point scale to rate their response from 0 (Not at all confident) to 4 (Completely confident). Scores for the 2 items are summed to create a total score. The PSEQ-2 was administered at baseline and on day 56. For pain self-efficacy with a VR referent, at baseline, participants rated their overall confidence in their ability to manage their pain on a 10-point scale from 1 (Not at all Confident) to 10 (Very Confident). Following the intervention, this section will be divided into 2 items measuring their overall confidence levels while inside VR and outside VR.

#### Chronic Pain Acceptance

The 8-item Chronic Pain Acceptance Questionnaire (CPAQ-8) short form is an 8-item validated instrument that assesses one’s engagement in personally meaningful activities despite pain, as well as efforts directed at controlling pain (example item: “I am getting on with the business of living no matter what my level of pain is”) [69]. Respondents rate each item using a 6-point scale ranging from 0 (never true) to 5 (always true).

#### Satisfaction With Treatment

Satisfaction with treatment was assessed with several items. First, using a 6-point scale (0=strongly disagree and 5=strongly agree), participants rated 4 items: ease of use of the VR headset, enjoyment of the headset, whether the headset helped with pain coping, and desire to continue using the VR headset. These 4 items were summed to create a total satisfaction score. Additionally, 1 item assessed likelihood to recommend VR (0=definitely not recommend and 10=definitely would recommend). One item assessed likelihood to continue using VR if they were able to keep their headset using a response scale (0=definitely would not it and 10=definitely would use it). Because of an error with the electronic survey administration, these data were captured at 1 month posttreatment.

#### VR Device Use

Device use data were recorded by the devices (date and time stamped for device access and duration of use).

#### System Usability Scale

The System Usability Scale (SUS) is a validated, 10-item scale to assess a global view system usability (example item: “I thought the system was easy to use”) [70]. Participants rate each item using a 5-point response scale ranging from 1=strongly disagree to 5=strongly agree. Some items are reverse scored, a multiplier is applied to the sum total, and total SUS scores range from 0-100.

#### Motion Sickness and Nausea (Cybersickness)

Adverse experiences with using VR was assessed using the question, “Did you experience any motion sickness or nausea while using VR?” on a 4-point scale, with 0=Never, 1=Sometimes, 2=Often, and 3=Always. Similar to prior work, cybersickness was assessed at the end of treatment [15]. Because of an error with the electronic survey administration, these data were captured at 1-month posttreatment.

#### Over-the-Counter Analgesic Medication Use

Participants were asked, “Do you take any ‘over the counter’ medication, meaning you can get yourself at a store without a prescription, to help you manage your back pain?” A binary response set (Y/N) was used to address variability in medication classes, formulations, doses, and frequency of use. Over-the-counter (OTC) analgesic use was measured at baseline and at posttreatment day 56.

#### Opioid Use Data

All opioid data were self-reported. Opioid medication doses were converted to a standardized morphine milligram equivalent daily dose using the Centers for Medicare & Medicaid Services “Opioid Oral Morphine Milligram Equivalent (MME) Conversion Table” [71]. Four assumptions were applied universally to all participants in calculating prescribed medication doses. First, participants who reported prescription medication use but did not report any of the classes of prescription medications were considered opioid free. Second, for participants who did not report the strength of their tablets, the most common dose of the tablet was used for the calculations (Hydrocodone 5 mg, Hydromorphone 2 mg, Oxycodone 5 mg, and Tramadol 50 mg); these doses are the lowest strength available for these medications and thus provide conservative estimates. Third, some participants reported using opioids “as needed” (ie, pro re nata [PRN] use) but did not detail their general frequency of use. For these cases, we calculated the dose and range based on 0 to maximum daily allowed (eg, for a participant prescribed medication every 6 hours PRN, we used the allowable range of 0-4 tablets per day and used the average value of 2 tablets per day). Fourth and last, if participants reported their frequency of opioid use to be weekly, the reported dose was divided by 7 for calculating a daily MME; similarly, monthly reported doses were divided by 30 to calculate a daily MME.

### Adverse Event Monitoring

Participants were provided with study staff contact information and encouraged to contact as needed and in the event of any problems using their device or with their treatment. Similar to other studies, cybersickness was assessed at the end of treatment [15]. However, due to a problem with the electronic survey, these data were not captured, and the survey was re-administered at 1-month posttreatment.

### Sample Size Determination

A power analysis was performed using data from a prior RCT of a 21-day at-home VR for chronic pain compared to an audio-only version of the treatment [15]. This study revealed that an average pre–post treatment difference score in pain intensity was 1.48 for the VR group and 0.756 for the audio-only group (on an 11-point scale). Assuming an α level of .05 and 90% power, 45 participants per group would detect a treatment group × time interaction. To buffer against potential high attrition (40%), a minimum of 75 participants would be required per treatment group, with 90 participants per treatment group being ideal.

### Statistical Analyses

All analyses involved 2-sided hypothesis tests, with α=.05 and adjusted for any multiple comparisons within the family of tests as appropriate. Group equivalence was assessed through univariate tests of association between treatment groups (EaseVRx/Sham VR) for all baseline demographic and clinical variables with chi-square and Kruskal–Wallis applied as appropriate.

The data were analyzed in a mixed-model framework (PROC GLIMMIX in SAS) using a marginal (population-averaged) model to allow for correlated responses across the repeated measures. There were 3 explanatory factors: treatment group, time, and time × treatment group. Treatment group, EaseVRx versus Sham VR, was specified as a fixed-effects factor. Time was specified as a random-effects factor to allow for correlated response using heterogeneous compound symmetry for the covariance structure within time. The 2 effects of interest were (1) the EaseVRx versus Sham VR between-group comparison across all time points, and (2) the time × treatment group effect which tests whether the treatment group influenced the trajectory of the key variables over time.

Data were 95.05% complete; missing values were not imputed for estimation of effects, but the predicted means were used in the graphical description. The primary outcomes were the time course of DVPRS (pain scale) from baseline (defined as the average of 3 pain ratings obtained during the 2 weeks before enrollment/randomization), at 8 weekly time points (twice per week) across the 8-week treatment period, and immediately posttreatment (Day 56). A linear mixed model was used with the treatment group (EaseVRx versus Sham VR) as an independent groups factor (ie, a between-subjects factor) and time of measurement as a dependent groups factor (ie, a within-subjects factor). DVPRS-II measures were analyzed using the same approach. Effect sizes for the EaseVRx versus Sham VR for the between-groups comparison were calculated using the standardized mean difference version of Cohen *d* [72]. The effect sizes for the within-subjects comparison (baseline to immediately posttreatment completion at day 56) were computed by treatment group using *d_rm_*, an adaptation of Cohen *d* to suit the repeated measures design [73].

## Results

### Study Participants

**Figure 3:**
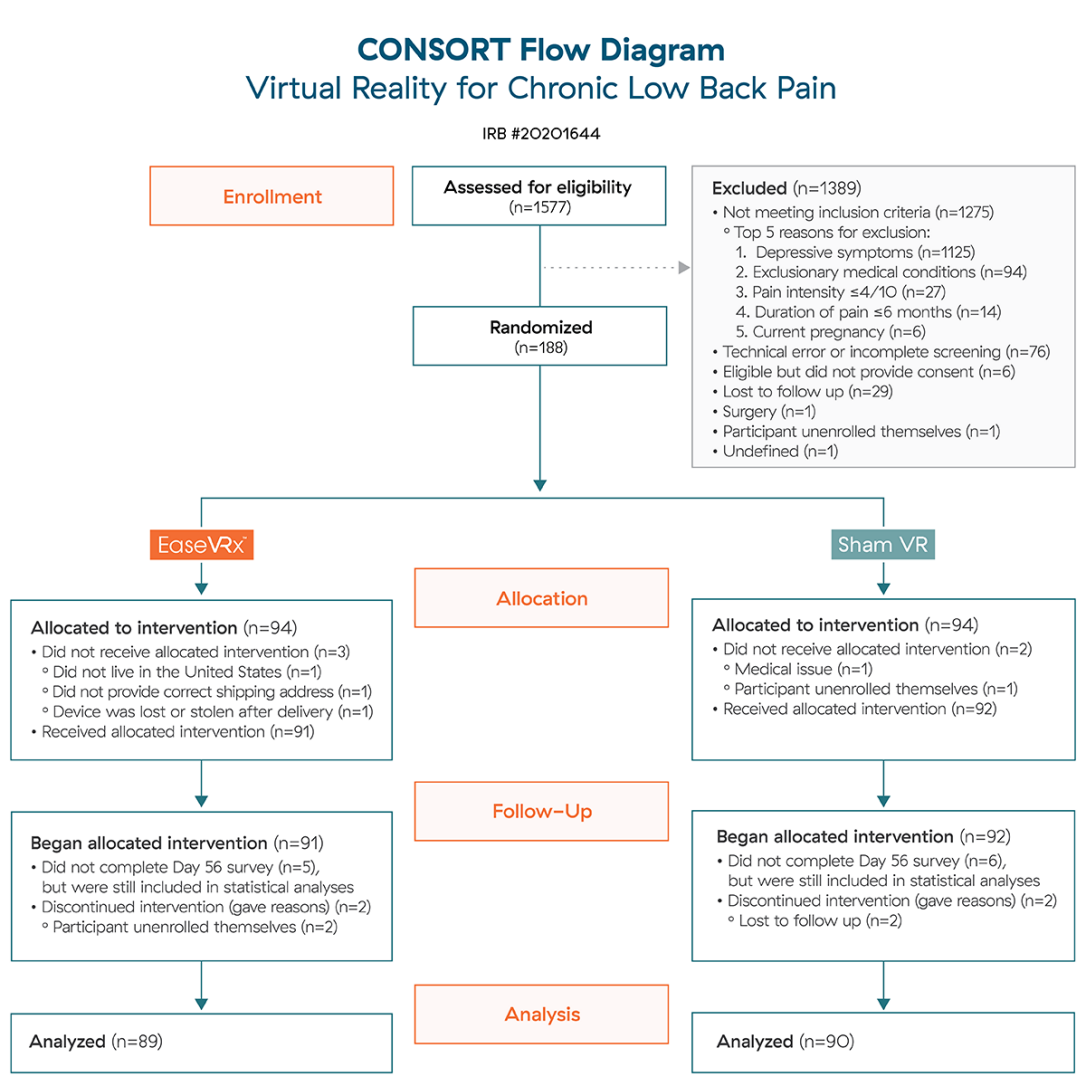
CONSORT Flow Diagram.

Figure 3 shows the CONSORT diagram for the study (see Multimedia Appendix 2 for the CONSORT checklist). In total, 1577 individuals were assessed for eligibility and 1389 were excluded with the primary reason of having met the threshold for depressive symptoms. A total of 188 individuals were enrolled, randomized, and allocated to the treatment group. After randomization, 9 individuals discontinued participation, 5 were unable to receive a VR device, 1 returned their unopened device due to a recent medical diagnosis, and 4 voluntarily withdrew for unknown reasons. A total of 179 individuals received a VR device with their assigned treatment (EaseVRx [n=89] or Sham VR [n=90]). Because intention-to-treat analyses were performed, the analytic data set includes 11 individuals who did not provide complete data. Nearly 94% of participants in group A (84/89) and 93% of participants in group B (84/90) completed the day 56 assessment (end of treatment).

Table 2 displays the baseline demographic characteristics by treatment group. The sample included 179 participants from 40 states. RUCA codes were applied to categorize participants by zip code. In total, 76.5% (n=137) resided in highly urban or metropolitan areas, 13.4% (n=24) participants resided in metropolitan or micropolitan areas, 9.5% (n=17) were from small town or rural areas, and 0.6% (n=1) had no rural–urban identifier information. The sample was predominantly female (76.5%, 137/179), Caucasian (90.5%, 162/179), with at least some college education (91.1%, 163/179), and a mean age of 51.5 years (SD 13.1; range 18-81). No significant between-group differences were observed for any demographic variable, thus demonstrating that randomization was effective.

Table 3 presents the baseline pain and clinical characteristics for the sample by treatment group. The sample duration of back pain was 5 or more years, and the mean pain intensity score was 5/10 (SD 1.2; range 1-9). No significant differences were observed between treatment groups for all variables assessed.

**Table 2:** Baseline demographic characteristics by treatment group.

Demographics		Treatment group		
		EaseVRx (N=89)	Sham VR^a^ (N=90)	*P*-value
**Gender, n (%)**				.527^b^
	Male	22 (25)	19 (21)	
	Female	67 (75)	70 (78)	
	Other	0 (0)	1 (1)	
**Age (years)**				.964^c^
	Mean (SD)	51.5 (13.5)	51.4 (12.9)	
	Range	18.0-81.0	25.0-81.0	
	Median (IQR)	51.0 (40.0-62.0)	54.0 (41.0-62.0)	
**Race, n (%)**				.247^b^
	Asian	2 (2)	1 (1)	
	Caucasian	78 (88)	84 (93)	
	African American	5 (6)	1 (1)	
	Multiracial	2 (2)	3 (3)	
	Other	2 (2)	0 (0)	
	Missing	0 (0)	1 (1)	
**Education, n (%)**				.142^b^
	High-school graduate	6 (7)	9 (10)	
	Some college	21 (24)	17 (19)	
	Associate	10 (11)	16 (18)	
	Undergraduate	17 (19)	25 (28)	
	Postgraduate	35 (39)	22 (24)	
	Missing	0 (0)	1 (1)	
**Employment status, n (%)**				.781^b^
	Part-time	9 (10)	7 (8)	
	Full-time	37 (42)	34 (38)	
	Not working	13 (15)	10 (11)	
	Retired	15 (17)	20 (22)	
	Unable to work	15 (17)	18 (20)	
	Missing	0 (0)	1 (1)	
**Annual household income, n (%)**				.665^b^
	<US $40,000	22 (25)	22 (24)	
	US $40,000-US $59,999	24 (27)	18 (20)	
	US $60,000-US $79,999	16 (18)	18 (20)	
	≥US $80,000	26 (29)	32 (36)	
	Missing	1 (1)	0 (0)	
**Marital** **status, n (%)**				.605^b^
	Married/Civil union	52 (58)	61 (68)	
	Divorced/Widowed/Separated	20 (22)	14 (16)	
	Single	10 (11)	10 (11)	
	Cohabitating	6 (7)	5 (6)	
	Missing	1 (1)	0 (0)	

^a^VR: virtual reality.

^b^Chi-square *P*-value.

^c^Kruskal–Wallis *P*-value.

**Table 3:** Baseline clinical variables by treatment group.^a^

Variables		Treatment group					
		EaseVRx (N=89)		Sham VR^b^ (N=90)		*P*-value	
**Pain duration, n (%)**						.082^c^	
	<1 year		7 (8)		1 (1)		
	1 year to <5 years		25 (28)		26 (29)		
	5 years to <10 years		15 (17)		24 (27)		
	>10 years		42 (47)		39 (43)		
**Average pain intensity**						.616^d^	
	Mean (SD)		5.1 (1.2)		5.2 (1.1)		
	Range		2.2-8.2		2.8-7.8		
	Median (IQR)		5.0 (4.2-5.8)		5.2 (4.4-5.6)		
**Pain interference with activity**						.398^d^	
	Mean (SD)		5.3 (1.8)		5.5 (1.5)		
	Range		1.2-10.0		1.0-8.8		
	Median (IQR)		5.6 (4.0-6.4)		5.5 (4.6-6.2)		
**Pain interference with mood**						.340^d^	
	Mean (SD)		4.5 (2.1)		4.7 (2.0)		
	Range		0.0-8.8		0.2-9.6		
	Median (IQR)		4.4 (2.8-5.8)		4.6 (3.6-5.8)		
**Pain interference with sleep**						.281^d^	
	Mean (SD)		4.8 (2.6)		5.3 (1.9)		
	Range		0.0-10.0		0.6-9.6		
	Median (IQR)		5.0 (3.0-7.0)		5.4 (3.8-6.4)		
**Pain interference with stress**						.852^d^	
	Mean (SD)		4.6 (2.2)		4.8 (2.0)		
	Range		0.0-10.0		0.6-9.6		
	Median (IQR)		4.8 (3.0-6.4)		5.0 (3.4-6.2)		
**Pain self-efficacy**						.766^d^	
	Mean (SD)		3.0 (1.5)		3.0 (1.2)		
	Range		0.0-6.0		0.0-6.0		
	Median (IQR)		3.0 (2.0-4.0)		3.0 (2.5-4.0)		
**Pain catastrophizing**						.977^d^	
	Mean (SD)		8.0 (3.8)		8.0 (3.5)		
	Range		0.0-16.0		0.0-16.0		
	Median (IQR)		8.0 (5.0-11.0)		7.0 (5.0-11.0)		
**Physical function**						.276^d^	
	Mean (SD)		38.3 (5.1)		37.6 (4.6)		
	Range		21.0-48.9		27.1-59.0		
	Median (IQR)		37.6 (35.0-41.2)		37.6 (35.0-40.2)		
**Chronic pain acceptance**						.708^d^	
	Mean (SD)		24.5 (7.3)		23.9 (6.7)		
	Range		5.0-42.0		7.0-47.0		
	Median (IQR)		24.0 (20.0-28.0)		23.0 (20.0-28.0)		
**Sleep disturbance**						.230^d^	
	Mean (SD)		56.7 (5.2)		57.6 (4.4)		
	Range		44.2-67.5		45.5-69.0		
	Median (IQR)		56.3 (53.3-60.4)		58.3 (55.3-60.4)		
Opioid use, n (%)		22 (25)		33 (37)		.083	
**Opioid dose (daily morphine milligram equivalent)**						.158^d^	
	Mean (SD)		25.2 (106.2)		15.3 (41.1)		
	Range		0.0-875.0		0.0-300.0		
	Median (IQR)		0.0 (0.0-0.0)		0.0 (0.0-10.0)		
OTC analgesic use, n (%)		61 (69)		55 (61)		.320	

^a^Baseline for the 5 pain variables (pain intensity, pain-related activity, mood, sleep, and stress interference) represents the average from 5 administrations in the pretreatment phase (days –14, –10, –7, –3, and 0).

^b^VR: virtual reality.

^c^Chi-square *P*-value.

^d^Kruskal–Wallis *P*-value.

### Treatment Engagement

Device use data revealed nonsignificant between-group differences for treatment engagement: EaseVRx participants completed a total of 43.30 (SD 15.91) experiences (average 5.4 per week) and Sham VR participants completed 48.06 (SD 24.78) experiences (average 6.0 per week).

### Device Safety and Adverse Events

Of the 147 participants who completed the 1-month posttreatment survey, 7/72 (9.7%) from the EaseVRx group and 5/75 (6.7%) from the Sham VR group reported experiencing nausea and motion sickness during the treatment phase of the study (*P*=.50). Participants were encouraged to contact study staff with any problems experienced during treatment; however, no participants contacted study staff to report adverse events of any type, including nausea and motion sickness.

### Primary Outcomes

**Figure 4:**
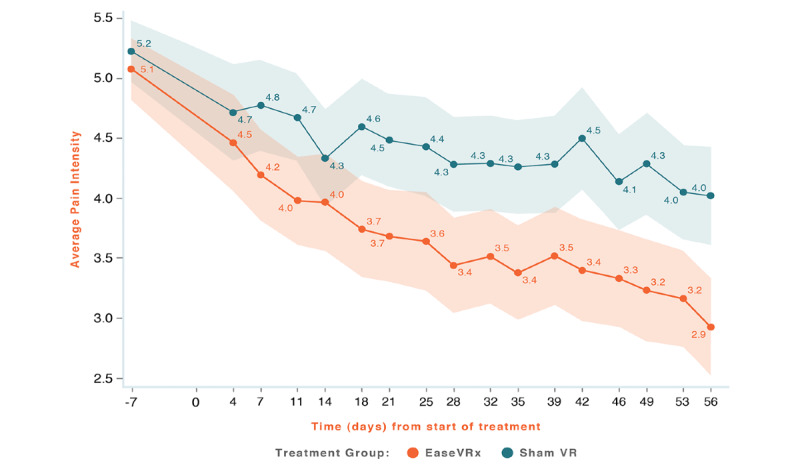
Average pain intensity.

**Figure 5:**
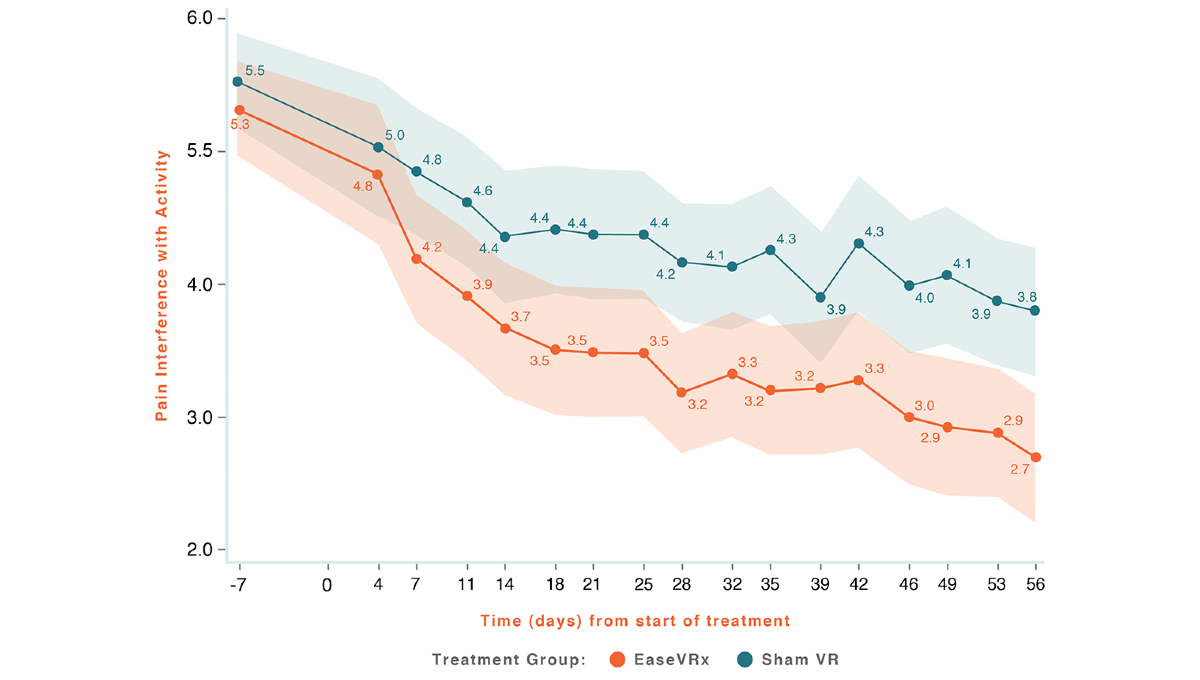
Pain-Related Interference with Activity.

**Figure 6:**
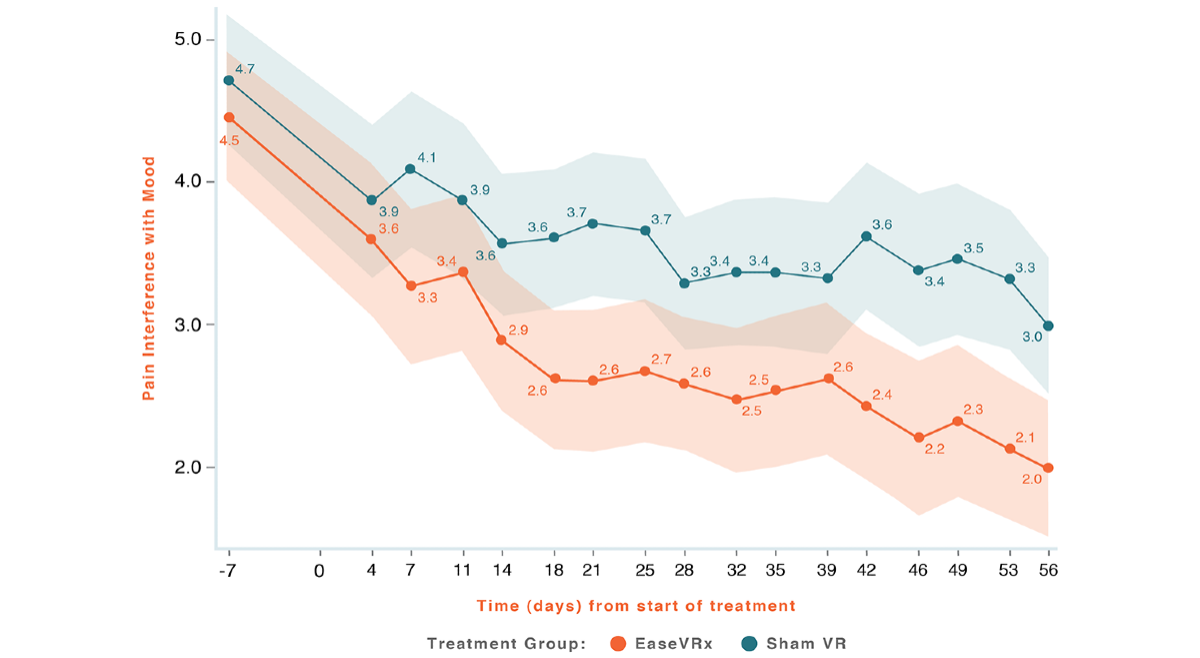
Pain-Related Interference with Mood.

**Figure 7:**
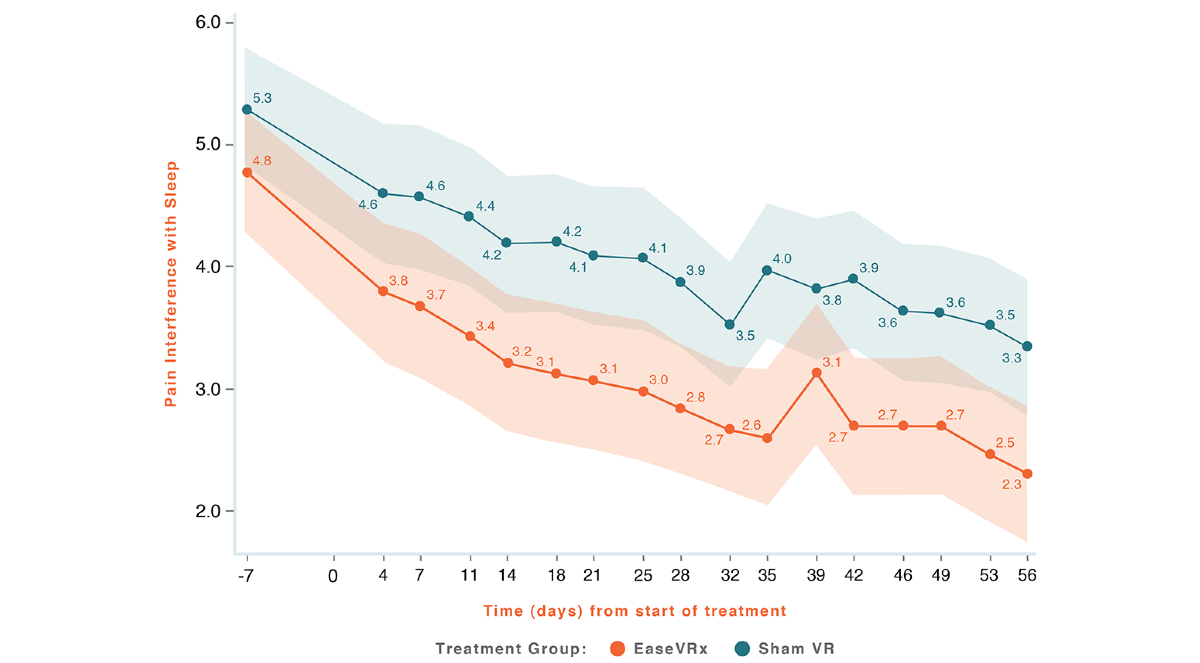
Pain Related Interference with Sleep.

**Figure 8:**
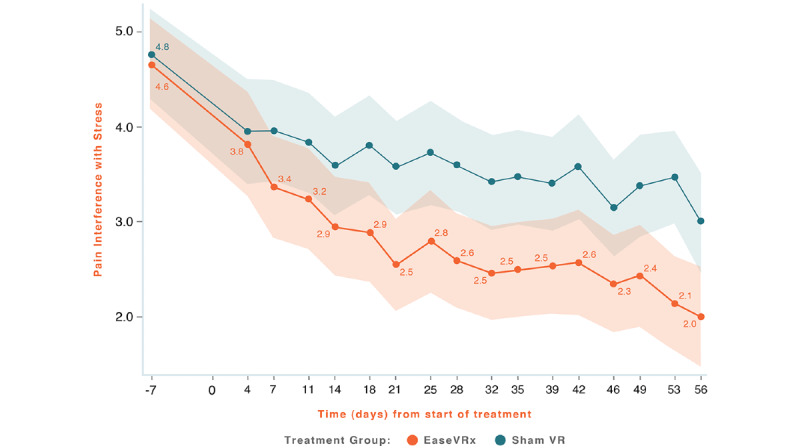
Pain-Related Interference with Stress.

Common analyses and data visualization were applied for all primary outcomes. The x-axis represents time (days), with days –14 to 0 averaged and labeled “day 7” to represent the pretreatment phase, days 1-55 were the active treatment phase, and day 56 was the end of treatment and the primary endpoint. The color bands represent the 95% CI values for the mean after correcting for multiple comparisons (Tukey–Kramer). Overlapping bands indicate nonsignificant treatment group differences (*P*-value) of simple main effects within each time point. The corresponding model effects for each primary outcome are displayed in Figures 4-8.

We observed a significant treatment effect (*P*=.001); on average, the EaseVRx group had lower pain intensity compared to the Sham VR group (Cohen *d*=0.49). Separately, we observed a time effect; average pain intensity significantly decreased over time for both treatment groups (time effect, *P*<.001). Most importantly, the decrease was greater for EaseVRx versus Sham VR (treatment × time effect, *P*<.001). Pain intensity reduced by an average of 42.8% for the EaseVRx group and 25.1% for the Sham VR group. The *d_rm_* for EaseVRx was 1.31, with combined results showing large effect size and moderate clinical importance. The VR Sham group *d_rm_* was 0.75, with combined results showing a large effect size and minimal clinical importance. As much as 65% (55/84) of EaseVRx and 40% (34/84) of Sham VR participants achieved 30% or more reduction in pain intensity. For EaseVRx, 46% (39/84) achieved 50% or more pain reduction, while for Sham VR 26% (22/84) reached that threshold.

We observed a significant treatment effect (*P*=.004) on pain interference with activity; on average, the EaseVRx group had lower activity interference compared to the Sham VR group (Cohen *d*=0.44). We also observed a time effect; pain interference with activity decreased over time for both treatment groups (time effect, *P*<.001). Most importantly, the decrease was greater for EaseVRx versus Sham VR (treatment × time effect, *P*=.013). Pain interference with activity reduced by an average of 51.6% for the EaseVRx group and 32.4% for the Sham VR group. The *d_rm_* for the EaseVRx group was 1.27, with combined results showing large effect size and moderate clinical importance. As much as 71% (60/84) of EaseVRx and 57% (48/84) of Sham VR participants achieved 30% or more reduction in pain-interference with activity, and 56% of (47/84) of the EaseVRx participants achieved 50% or more reduction. The VR Sham group *d_rm_* was 0.97, with combined results showing a large effect size and moderate clinical importance.

We observed a significant treatment effect (*P*=.005) on pain interference with mood; on average, the EaseVRx group had lower mood interference compared to the Sham VR group (Cohen *d*=0.42). We also observed a time effect; pain-interference with mood decreased over time for both treatment groups (time effect, *P*<.001) and the decrease was greater for EaseVRx versus Sham VR (treatment × time effect, *P*=.010). Pain interference with mood reduced by an average of 55.7% for EaseVRx and 40.04% for the Sham VR. The *d_rm_* for the EaseVRx was 1.18, with combined results evidencing a large effect size and substantial clinical importance. As much as 74% (62/84) of EaseVRx participants and 60% (50/84) of Sham VR participants achieved 30% or more reduction in pain-related interference with mood, and 61% (51/84) of the EaseVRx participants achieved 50% or more reduction. The *d_rm_* for the VR Sham group was 0.79, with combined results showing a moderate effect size and moderate clinical importance.

We observed a significant treatment effect (*P*=.004) on pain-interference with sleep; on average, the EaseVRx group had lower sleep interference compared to the Sham VR group (Cohen *d*=0.44). We also observed a time effect; pain-interference with sleep decreased over time for both treatment groups (time effect, *P*<.001). However, there was no difference between treatment groups over time (*P*=.755). Pain interference with sleep reduced by an average of 54% for EaseVRx and 39.2% for the Sham VR. The *d_rm_* for the EaseVRx was 0.95, with combined results showing large effect size and substantial clinical importance for symptom reduction. As much as 70% (59/84) of EaseVRx and 60% (50/84) of participants achieved 30% or more reduction in Pain-related interference with sleep, and 60% (50/84) of the EaseVRx participants achieved 50% or more reduction. The VR Sham group *d_rm_* was 0.87, with combined results showing a large effect size and moderate clinical importance.

We observed a significant treatment effect (*P*=.009) on pain interference with stress; on average, the EaseVRx group had lower stress interference compared to the Sham VR group (Cohen *d*=0.40). We also observed a time effect; pain interference with stress decreased over time for both treatment groups (time effect, *P*<.001). Most importantly, the decrease was greater for EaseVRx versus Sham VR (treatment × time effect, *P*=.004). Pain interference with stress reduced by an average of 59.9% for the EaseVRx group and 38.3% for the Sham VR group. The *d_rm_* for the EaseVRx was 1.17, with combined results evidencing a large effect size and substantial clinical importance. As much as 76% (64/84) of EaseVRx participants and 56% (47/84) of the Sham VR participants achieved 30% or more reduction in pain-related interference with stress, and 63% (53/84) achieved 50% or more reduction. The VR Sham group *d_rm_* was 0.77, with combined results showing a large effect size and moderate clinical importance.

### Secondary Outcomes

#### Treatment Engagement

Device use data were received for 149 participants (EaseVRx =77; Sham VR = 72). EaseVRx participants completed a mean of 43.3 (SD 15.9) sessions, while the Sham VR group completed a mean of 48.1 (SD 24.8) sessions. A significant group difference for treatment engagement was not found.

#### Patient’s Global Impression of Change

##### Between-Subjects Analysis

The between-subjects analysis of PGIC at posttreatment indicated a significant effect of condition (*P*=.002); participants in the EaseVRx group reported greater PGIC than those in the Sham VR group (4.13 versus 3.11).

While both groups evidenced improvement in pain coping symptoms, including pain catastrophizing, pain self-efficacy, and pain acceptance from pretreatment to end of treatment, none of these improvements achieved statistical significance.

##### Physical Function

For PROMIS Physical Function, we observed a significant treatment effect (*P*=.022); the EaseVRx group had higher physical function compared to the Sham VR group (Cohen *d*=0.34). Both treatment groups significantly improved from baseline to postintervention; there was superior functional improvement for the EaseVRx group relative to the Sham VR group (time × condition effect, *P*=.002). The *d_rm_* values for the EaseVRx and VR Sham groups were 0.64 and 0.35, respectively.

##### Sleep Disturbance

For PROMIS sleep disturbance, we observed a significant treatment effect (*P*=.013); the EaseVRx group had lower sleep disturbance compared to the Sham VR group (Cohen *d*=0.37). Both treatment groups significantly improved throughout the study; there was superior improvement for the EaseVRx group relative to the Sham VR group (time × condition effect, *P*=.035). The *d_rm_* for the EaseVRx group was 0.83, evidencing a large effect and substantial clinical importance.

#### Prescription Opioid and OTC Analgesic Use

Neither treatment group evidenced a significant change in MME dose from baseline to end of treatment. For OTC analgesic medication use, a substantial decrease was observed in the EaseVRx group. While 61 reported using OTC analgesics at baseline, 50 reported use at posttreatment day 56 (*P*=.01). For Sham VR, 55 and 56 reported OTC analgesic use at baseline and posttreatment, respectively (nonsignificant).

#### Treatment Satisfaction, Likelihood to Recommend, and Likelihood to Continue Use

For the 4 summed satisfaction items, the EaseVRx group reported greater satisfaction with treatment than the Sham VR group (4.32 versus 3.46 respectively; *P*<.001). Similarly, the EaseVRx group reported greater likelihood to recommend VR to someone else compared to the Sham VR group (8.72 versus 6.55, respectively; *P*<.001). Finally, EaseVRx participants reported greater likelihood to continue using VR if they could keep their headset compared to Sham VR (9.18 versus 7.23, respectively; *P*<.001).

#### VR Usability Ratings

Both treatment groups reported high usability with no statistical difference between groups (EaseVRx usability rating = 84.33; Sham VR usability rating = 81.16).

#### Additional Analyses

Two additional Sham VR participants provided data for only 1 and 2 of the 16 surveys; including them in the analysis (for a total of 92) did not alter the significance of the study findings for any variable at any time point.

## Discussion

### Principal Findings

We conducted the first placebo-controlled RCT of home-based therapeutic VR in a national sample of individuals with cLBP. We hypothesized that an 8-week pain relief skills VR program (EaseVRx) would be superior to Sham VR at posttreatment (day 56) for our primary outcomes: average pain intensity and pain-related interference with activity, mood, sleep, and stress. While both study groups had significant reductions in pain and all domains of pain-related interference, EaseVRx evidenced superior treatment effects for all primary outcomes except sleep interference; the between-groups Cohen *d* effect sizes ranged from 0.40 to 0.49. For EaseVRx, large pre/posttreatment Cohen *d* effect sizes ranged from 1.17 to 1.3 and demonstrated moderate to substantial clinical importance for reduced pain intensity and pain-related interference with activity, mood, and stress at end of treatment. A greater proportion of participants in the EaseVRx group exceeded thresholds for clinical importance of effects. For moderate clinical importance in pain reduction (≥30% reduction in pain), 65% (55 of 84) in EaseVRx versus 40% (34 of 84) in Sham VR met this threshold. For substantial clinical importance in pain reduction (≥50% reduction in pain), 46% (39 of 84) in EaseVRx versus 26% (22 of 84) in Sham VR met this threshold. These effects for therapeutic VR exceeded effects reported for a 3-week skills-based VR program for chronic pain [15] and effects reported for CBT studies involving in-person 8-week treatment with a trained therapist [4,6,7].

Both treatment groups evidenced moderate to substantial reductions for pain-related interference with sleep; however, no between group differences were found. By contrast, EaseVRx was superior to Sham VR for reducing sleep disturbance (secondary outcome), suggesting that therapeutic VR is particularly effective for reducing general versus pain-related sleep disturbance.

The secondary outcomes yielded interesting findings. First, physical function significantly improved for both treatment groups, with superior improvements found for EaseVRx. We note that the therapeutic content included no kinematic elements, nor did it include direction for activity, movement, or goal setting for either. Accordingly, improvement in physical function may be a product of substantially reduced pain-related interference in activity. Next, our hypothesis that therapeutic VR would be superior to Sham VR for improving pain coping (eg, reducing pain catastrophizing and improving pain self-efficacy and chronic pain acceptance) was unmet. The lack of effect for pain catastrophizing was particularly striking because this is a malleable construct that is highly responsive to behavioral treatments broadly [4,6,7] (Darnall et al, unpublished). However, our results align with the prior 3-week VR program study that similarly found no effect on pain catastrophizing and pain self-efficacy. Findings suggest that increased treatment time and additional focal content were insufficient to affect these factors significantly. These findings also highlight differences in treatment efficacy between therapeutic VR and CBT. Multiple studies have shown that CBT imparts its largest effect on pain catastrophizing [4]. By contrast, therapeutic VR evidenced its largest effects for reducing pain intensity and pain interference across several key domains.

We found no differences for either treatment group for change in prescription opioid use, and note that prescribing changes are unlikely to occur within the 2-month timeframe of the study. Similarly, we found no changes in “as needed” opioid use. We found substantially reduced use of OTC analgesic medication at posttreatment for the EaseVRx group only. While additional research is needed to replicate this finding, this finding offers a promising suggestion that therapeutic VR may reduce need for analgesics. Future VR research should examine medication use in greater detail and with higher-frequency data capture.

The study’s methodologic rigor was strengthened by a placebo treatment (Sham VR), which evidenced equivalent participant engagement as therapeutic VR. The extant literature on digital behavioral health research has reported participant treatment engagement rates ranging from 20% to 60% [13,74-76]. Strikingly, the current trial evidenced a 90% engagement rate in both groups, thus suggesting that efforts to enhance the face validity of the Sham VR were effective. These results also highlight the public interest in home-based VR as a chronic pain treatment modality. Therapeutic VR was rated significantly higher than Sham VR for satisfaction, likelihood to recommend to others, and likelihood to continue using the device after the 8-week treatment phase if it was made available. Combined with high participant engagement data and device usability ratings, these data extend prior work [15] supporting the utility, user satisfaction, and efficacy of home-based VR for chronic pain.

The context of the COVID-19 pandemic may have influenced the participant engagement rate. This trial occurred entirely at a time when people were adhering to strict social distancing measures and were environmentally isolated. Indeed, for many people receipt of medical care is worryingly low due to limited availability or unavailability of outpatient treatment options. These circumstances likely supported interest in effective home-based pain care. The COVID-19 context and the home-based study design support the ecological validity of the study findings. Notably, the study was conducted remotely and did not benefit from any in-person contacts or enhanced placebo effects that occur when research involves high-touch protocols or is conducted in medical treatment settings (ie, halo effects).

Strengths of this study include methods that attended to the IMMPACT recommendations and the NIH Research Standards for Back Pain. The study was conducted in a national sample drawn from 40 states, was well-distributed geographically, and included participants from urban and rural settings. Additional aspects of methodological rigor included participant and analyst blinding, intention-to-treat analyses, randomization, and a rigorous placebo control group that adhered to recommended specifications for an optimal VR sham [77].

### Limitations

Several limitations bear consideration when evaluating the study results. With the exception of device use metrics, all data were self-reported. The study was untethered from medical care and thus, there was no ability to confirm pain diagnoses or analgesic prescription information. The study sample was predominantly female, white, college educated, and internet savvy; thus, findings may not generalize to individuals with disparate demographic characteristics. These findings are consistent with previous evidence showing that highly educated females are more likely to use self-care mobile health technologies, particularly those with mindfulness-based content [78], and this dovetails with a general female predilection to seek treatment for pain and other health concerns. As eHealth literacy and awareness increase in clinicians and the general population, it is likely that health technologies (including therapeutic VR) will benefit other demographics [79]. Additionally, the study was conducted in individuals with cLBP and findings may not generalize to other chronic pain conditions.

Data on cybersickness were collected at 1 month posttreatment and this lag introduces potential for recall bias, despite others documenting that participants readily recall cybersickness due to its specificity and salience [15]. Overall attrition was low (n=11) and it is possible that the 2 EaseVRx participants who left the study after receiving their headset did so due to cybersickness. No participants contacted study staff to report adverse events of any type. Data on sex differences for cybersickness are mixed, with some reporting a female preponderance, while a recent meta-analysis suggests no sex effect [80]. Within the context of our limitations, we highlight low reports of cybersickness in a predominantly female sample. Future research may better capture potential VR adverse effects by assessing these factors in the first week of treatment. Interpretation of data on prescription opioid use was limited by low-frequency sampling methods that are subject to recall bias and poor data accuracy. Opioid prescriptions often allow “as needed” flexibilities in medication use and future research designs may benefit from high-frequency sampling methods which improve data accuracy. Further, in quantifying analgesic medication use (prescription opioids and OTC analgesics) we did not assess or control for life events or circumstances that may have influenced medication use (eg, acute injury or surgery). Finally, our threshold for depression screening and inclusion was applied to provide greater specificity [44] yet is noted to be lower than what is reported for many individuals with chronic pain. While our purpose in applying a low threshold for depressive symptoms was to minimize poor engagement and attrition resulting from anhedonia or avolition (and therefore poor data quality to determine treatment efficacy), recent research in cLBP suggests such concerns may be unfounded for mild to moderate depressive symptoms (Darnall et al, unpublished).

As a fully self-administered and on-demand treatment, VR is a promising and effective option that can transcend many traditional barriers to nonpharmacologic pain treatment; however, currently access is limited. With future reimbursement and commercial availability, therapeutic VR could become affordable and widely accessible to consumers.

### Conclusion

An 8-week self-administered home-based pain relief skills VR program appears effective for reducing pain intensity and pain-related interference in activity, mood, and stress posttreatment. Treatment effects ranged from moderately to substantially clinically important. Therapeutic VR had high rates for engagement and user satisfaction. Additional studies are needed to determine effects in demographically diverse populations and in other pain conditions. Data suggest therapeutic VR is not operating through traditional pain coping mechanisms. As such, additional research is needed to characterize mechanisms of treatment effects and durability of effects. Home-based VR appears to provide effective and on-demand nonpharmacologic treatment for cLBP.

## Appendix

Multimedia Appendix 1 eConsent form.

Multimedia Appendix 2 CONSORT-EHEALTH checklist (V.1.6.1).

## Abbreviations

CBT: cognitive behavioral therapy
cLBP: chronic low back pain
CPAQ-8: 8-item Chronic Pain Acceptance Questionnaire
DVPRS: Defense and Veterans Pain Rating Scale
MME: morphine milligram equivalent
OTC: over the counter
PCS: Pain Catastrophizing Scale
PGIC: Patient’s Global Impression of Change
PROMIS: Physical Function and Sleep Disturbance
PSEQ-2: 2-item Pain Self-Efficacy Questionnaire
RCT: randomized controlled trial
RUCA: rural–urban commuting area
SUS: System Usability Scale
VR: virtual reality
