# Supplementary material for: An 8-Week Self-Administered At-Home Behavioral Skills-Based Virtual Reality Program for Chronic Low Back Pain: Double-Blind, Randomized, Placebo-Controlled Trial Conducted During COVID-19
Source: J Med Internet Res. 2021 Feb 22;23(2):e26292. doi: 10.2196/26292 (PMC7939946; doi:10.2196/26292)
Supplement: Multimedia Appendix 1 [file jmir_v23i2e26292_app1.pdf]

AppliedVR
1840 Century Park East, #801, Los Angeles CA 90067
844-204-9093

IRB APPROVED
Jul 02, 2020

RESEARCH PARTICIPANT INFORMATION AND CONSENT FORM

**TITLE:**	Virtual Reality Trial Using EaseVRx For Chronic Low Back Pain
**PROTOCOL NO.:**	Pro000XXXX<br>WIRB® Protocol # 20201644
**SPONSOR:**	AppliedVR, Inc.
**INVESTIGATOR:**	Brandon Birckhead MD<br>1840 Century Park East, 8th floor<br>Century City, CA 90067<br>United States
**PHONE NUMBER(S):**	911 for emergency<br>24-hour Number - 844-204-9093<br><br>Laura Garcia – 310-890-0498<br><br>Dr. Brandon Birckhead, MD – 310-880-6853

WHAT IS THE PURPOSE OF THE STUDY?

You are invited to participate in a study being conducted by AppliedVR. The purpose of the study is to evaluate the effectiveness of an 8-week digital health wellness program in helping you manage chronic lower back pain and improve your quality of life with pain. As a participant in this study, you will experience a digital health program using a virtual reality headset. All participants will complete surveys to help researchers better understand their pain. The virtual reality device is an experimental form of pain treatment. The device and software pose minimal risks to participants.

The study will enroll up to 150 people in total.

WHAT WILL HAPPEN DURING THE STUDY?

This section provides a general overview of what will happen during your participation in this study.

This is a single-blind, randomized research study with a two-week screening period before randomization. Your study participation will not dictate your treatment or care.

- “Single-blind” means that the researchers will know which group you are assigned to but will not tell you which group you are participating in.
- “Randomized” means that you will be assigned to a study group by chance, like flipping a coin. You will be randomized into one of two study groups and will have an equal chance of being placed in one of the two groups described below.
- “Screening week” means you will receive surveys every 2-3 days for a week via email. Once you complete this week of surveys you will be eligible to be randomized.

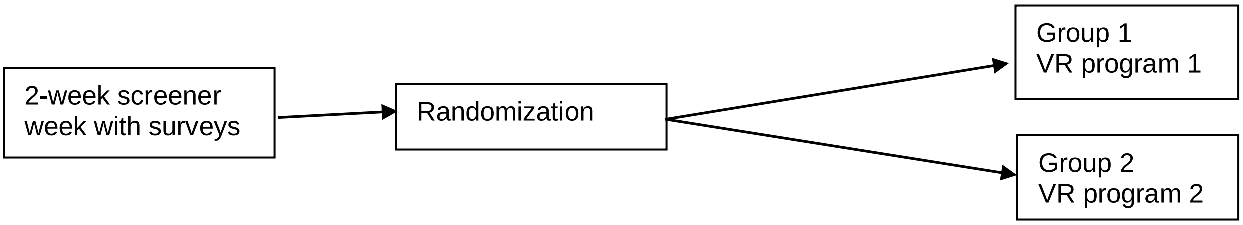

After you agree to participate in the study, you will be asked to complete surveys every two to three days to measure your pain levels. Before starting the digital health program, you will be asked to complete a short online questionnaire which will ask you about your current health. After you complete the baseline set of surveys, you will be randomized into one of two groups with different VR programs.

A computer will randomly assign you to a study group. This is done because no one knows if the results experienced by the participants in one study group are better, the same, or worse than the results experienced by participants in the other. Once you are put in one group, you cannot switch to another group. You cannot choose the group in which you will be placed.

You will receive a VR headset through the mail with the digital health program. You will receive an email with information to set up your device. Once your device is working, you will be asked to complete one VR session each day for eight weeks. Each session will last between 3-15 minutes.

During each VR session, you will be asked to wear a device, the PICO G2, with preloaded visualization software. You will be asked to complete surveys online sent via email or text twice a week over the eight weeks of the program. Once you finalize our program, we will ask you to complete surveys online at four weeks, eight weeks, 12 weeks, and 24 weeks post-study.

Participation in the study will entail eight weeks with the VR headset and 24 weeks of follow-up.

RISKS AND DISCOMFORTS

<u>Virtual Reality Intervention</u>
Participants using the virtual reality intervention may experience side effects common to users of VR and individuals who view 3D videos, including motion sickness, dizziness, eye strain, headaches, or other visual abnormalities. If participants experience these symptoms while using the device at home, they should stop using the device for 15 minutes and then attempt to resume the use of the device. If the symptoms do not resolve or recur and you are unable to continue the use of VR, you should contact the study team at the phone number listed on the first page. A small number of patients (up to 0.025%) may experience seizures or severe symptoms (e.g., disorientation, nausea, or drowsiness) upon viewing the virtual reality experience. Seizures from flashing light are more common in children and epileptic patients (who are excluded). To minimize this concern further, we have *not* incorporated flashing lights into the VR experiences. Some patients may find the VR goggles uncomfortable to wear or confining. To date, patients with claustrophobia have *not* reported discomfort using VR goggles. Nevertheless, individuals previously diagnosed with claustrophobia should discontinue use if they feel uncomfortable.

<u>Questionnaires:</u>
Some of the items in the surveys may make you feel uncomfortable or embarrassed. You are not required to respond to any item that you do not wish to answer. The surveys will be labeled with a unique study number that will link your identity so that only the research team can recognize you.

There are no anticipated long-term risks from participating in this study. There is the possible risk of loss of confidentiality of your research information. There may be unknown risks.

BENEFITS

If you agree to take part in this research study, there may or may not be direct medical benefit to you. The possible benefits of taking part in the research study are greater care satisfaction, pain management, and overall health during the study. However, no benefit is guaranteed. It is possible that your condition may remain unchanged or even get worse.

IRB APPROVED
Jul 02, 2020

We hope the information learned from this research study will benefit other patients by helping us learn whether patients enjoy using the VR interventions.

WHY WOULD MY PARTICIPATION BE STOPPED?

Your participation in this study may be stopped at any time by the researcher or the sponsor without your consent for any reason, including:

- The study is stopped or suspended;
- Support for the study is reduced, stopped or withdrawn;
- If it is in your best interest;
- You do not consent to continue in the study after being told of changes in the research that may affect you;
- You become pregnant; or
- You do not follow the study procedures.

ARE THERE ANY OTHER OPTIONS?

Other options for chronic pain relief include medication, cognitive behavior therapy, physical therapy, and relaxation techniques.

Your participation is voluntary, so you have the right to decline to participate or withdraw from this research study at any time without any penalty or loss of benefits to which you would otherwise be entitled outside of the study.

WILL MY INFORMATION BE KEPT CONFIDENTIAL?

Your survey answers will be stored initially with REDCap Cloud Electronic Data Collection (EDC) in a password protected electronic format. Data will later be downloaded and stored in a password-protected and encrypted Excel database which will have a participant number and initials and not have any personal identifiers associated with the survey responses.

We will do our best to make sure that your personal information collected as part of this study is kept private. However, we cannot guarantee total privacy. Your personal information may be given out if required by law. If information from this study is published or presented at scientific meetings, your name and other identifiable personal information will not be used. Organizations that may look at and/or copy your survey responses for research, quality assurance, and data analysis include: the Institutional Review Board, accrediting agencies, government and regulatory groups (such as Food and Drug Administration (FDA), Office for Human Research Protections (OHRP), etc.), safety monitors, companies that sponsor the study, and authorized representatives of the sponsor.

WHAT IF I BECOME ILL OR INJURED BECAUSE OF TAKING PART IN THIS STUDY?

Should you believe that you are ill or have been injured as a result of your participation, please contact the study team at the phone number listed on page 1 of this consent form. The study team will arrange for any treatment needed. No financial compensation for injury is routinely offered.

FINANCIAL CONSIDERATIONS

Costs of Participation
You will not be charged for your participation in this research study. The study sponsors will cover the cost of all items and services required by this study. You will be asked to return the virtual reality kit to AppliedVR at the completion of the study and be given a pre-paid shipping label to mail the kit back to AppliedVR.

IRB APPROVED
Jul 02, 2020

Compensation for Participating

You will receive up to $150 for completing the 56-day program and the post-intervention surveys administered after the initial 8-week program. Each participant's honorarium is based on the percentage of surveys completed. Those who complete 20%-40% of the surveys will be awarded $20. Those who complete 41%-60% of the surveys will be awarded $40. Those who complete 61%-80% of the surveys will be awarded $60. Those who complete greater than 80% of the surveys will be awarded $150 and will be entered into a raffle for a 1-in-10 chance to receive a VR headset with an AppliedVR solution installed.

Your compensation will be in the form of two Amazon gift codes sent to your email address. Your first payment will be sent immediately after your complete the 56-day program and return your headset with the shipping label we provide. Your second payment will be sent immediately after the last follow-up survey.

WHAT IF I HAVE QUESTIONS OR PROBLEMS?

Please contact one of the investigators listed on the first page of this form if you have a research-related problem or if you have questions, complaints, or concerns about the research.

If you have questions about your rights as a research participant, general questions, complaints, problems, or concerns that you want to discuss with someone who is not associated with this study, or want to offer suggestions or feedback, please contact:

Western Institutional Review Board® (WIRB®)
1019 39th Avenue SE Suite 120
Puyallup, Washington 98374-2115
Telephone: 1-800-562-4789 or 360-252-2500
E-mail: Help@wirb.com

WIRB is a group of people who independently review research.

WIRB will not be able to answer some study-specific questions, such as questions about appointment times. However, you may contact WIRB if the research staff cannot be reached or if you wish to talk to someone other than the research staff.

A description of this clinical trial will be available on http://www.ClinicalTrials.gov, as required by U.S. Law. This Web site will not include information that can identify you. At most, the Web site will include a summary of the results. You can search this Web site at any time.

AppliedVR
1840 Century Park East, #801, Los Angeles CA 90067
844-204-9093

IRB APPROVED
Jul 02, 2020

ELECTRONIC CONSENT

If you sign this form below, it indicates that:

- You have taken the time to carefully read and understand the information presented in this informed consent form;
- You have considered the potential risk and any anticipated benefits of participation as described in this consent form;
- You voluntarily agree to participate in this research study;
- You are 18 years of age or older

☐ Agree

☐ Disagree

NAME:

ELECTRONIC SIGNATURE VIA REDCap Cloud eConsent:

DATE:

We will email you an electronic copy of this signed and dated consent form to keep for your records.
