# Supplementary material for: An 8-Week Self-Administered At-Home Behavioral Skills-Based Virtual Reality Program for Chronic Low Back Pain: Double-Blind, Randomized, Placebo-Controlled Trial Conducted During COVID-19
Source: J Med Internet Res. 2021 Feb 22;23(2):e26292. doi: 10.2196/26292 (PMC7939946; doi:10.2196/26292)
Supplement: Multimedia Appendix 2 [file jmir_v23i2e26292_app2.pdf]

vCONSORT-EHEALTH (V 1.6.1) - Submission/Publication Form

The CONSORT-EHEALTH checklist is intended for authors of randomized trials evaluating web-based and Internet-based applications/interventions, including mobile interventions, electronic games (incl multiplayer games), social media, certain telehealth applications, and other interactive and/or networked electronic applications. Some of the items (e.g. all subitems under item 5 - description of the intervention) may also be applicable for other study designs.

The goal of the CONSORT EHEALTH checklist and guideline is to be

a) a guide for reporting for authors of RCTs,

b) to form a basis for appraisal of an ehealth trial (in terms of validity)

CONSORT-EHEALTH items/subitems are MANDATORY reporting items for studies published in the Journal of Medical Internet Research and other journals / scientific societies endorsing the checklist.

Items numbered 1., 2., 3., 4a., 4b etc are original CONSORT or CONSORT-NPT (non-pharmacologic treatment) items.

Items with Roman numerals (i., ii, iii, iv etc.) are CONSORT-EHEALTH extensions/clarifications.

As the CONSORT-EHEALTH checklist is still considered in a formative stage, we would ask that you also RATE ON A SCALE OF 1-5 how important/useful you feel each item is FOR THE PURPOSE OF THE CHECKLIST and reporting guideline (optional).

Mandatory reporting items are marked with a red *.

In the textboxes, either copy & paste the relevant sections from your manuscript into this form - please include any quotes from your manuscript in QUOTATION MARKS,

or answer directly by providing additional information not in the manuscript, or elaborating on why the item was not relevant for this study.

YOUR ANSWERS WILL BE PUBLISHED AS A SUPPLEMENTARY FILE TO YOUR PUBLICATION IN JMIR AND ARE CONSIDERED PART OF YOUR PUBLICATION (IF ACCEPTED).

Please fill in these questions diligently. Information will not be copyedited, so please use proper spelling and grammar, use correct capitalization, and avoid abbreviations.

DO NOT FORGET TO SAVE AS PDF _AND_ CLICK THE SUBMIT BUTTON SO YOUR ANSWERS ARE IN OUR DATABASE !!!

Citation Suggestion (if you append the pdf as Appendix we suggest to cite this paper in the caption):

Eysenbach G, CONSORT-EHEALTH Group

CONSORT-EHEALTH: Improving and Standardizing Evaluation Reports of Web-based and Mobile Health Interventions

J Med Internet Res 2011;13(4):e126

URL: http://www.jmir.org/2011/4/e126/

doi: 10.2196/jmir.1923

PMID: 22209829

* Required

Your name *

Beth Darnall

Primary Affiliation (short), City, Country *

Department of Anesthesiology, Perioperative and Pain Medicine, Stanford University School of Medicine, Stanford, CA, United States.

Your e-mail address *

abc@gmail.com

bdarnall@stanford.edu

Title of your manuscript *

Double-blind, pragmatic, randomized placebo-controlled trial of 8-week self-administered at-home behavioral skills-based virtual reality (VR) for chronic low back pain

Name of your App/Software/Intervention *

EaseVRx

Evaluated Version (if any)

e.g. "V1", "Release 2017-03-01", "Version 2.0.27913"

NA

Language(s) *

What language is the intervention/app in? If multiple languages are available, separate by comma (e.g. "English, French")

English

URL of your Intervention Website or App

e.g. a direct link to the mobile app on app in appstore (itunes, Google Play), or URL of the website. If the intervention is a DVD or hardware, you can also link to an Amazon page.

Your answer

URL of an image/screenshot (optional)

Your answer

Accessibility *

Can an enduser access the intervention presently?

access only for special usergroups, not open

Primary Medical Indication/Disease/Condition *

e.g. "Stress", "Diabetes", or define the target group in brackets after the condition, e.g. "Autism (Parents of children with)", "Alzheimers (Informal Caregivers of)"

Lower Back Chronic Pain

Primary Outcomes measured in trial *

comma-separated list of primary outcomes reported in the trial

change in average pain intensity, pain-related interference with activity, stress, mood, sleep

Secondary/other outcomes

Are there any other outcomes the intervention is expected to affect?

global impression of change, physical function, sleep disturbance, pain self-efficacy, pain catastrophizing, pain acceptance, pain relief skills use, pain medication use, user satisfaction

Recommended "Dose" *

What do the instructions for users say on how often the app should be used?

Approximately Daily

Approximately Weekly

Approximately Monthly

Approximately Yearly

"as needed"

Other:

Approx. Percentage of Users (starters) still using the app as recommended after 3 months *

unknown / not evaluated

0-10%

11-20%

21-30%

31-40%

41-50%

51-60%

61-70%

71%-80%

81-90%

91-100%

Other:

Overall, was the app/intervention effective? *

yes: all primary outcomes were significantly better in intervention group vs control

partly: SOME primary outcomes were significantly better in intervention group vs control

no statistically significant difference between control and intervention

potentially harmful: control was significantly better than intervention in one or more outcomes

inconclusive: more research is needed

Other:

Article Preparation Status/Stage *

At which stage in your article preparation are you currently (at the time you fill in this form)

not submitted yet - in early draft status

not submitted yet - in late draft status, just before submission

submitted to a journal but not reviewed yet

submitted to a journal and after receiving initial reviewer comments

submitted to a journal and accepted, but not published yet

published

Other:

Journal *

If you already know where you will submit this paper (or if it is already submitted), please provide the journal name (if it is not JMIR, provide the journal name under "other")

not submitted yet / unclear where I will submit this

Journal of Medical Internet Research (JMIR)

JMIR mHealth and UHealth

JMIR Serious Games

JMIR Mental Health

JMIR Public Health

JMIR Formative Research

Other JMIR sister journal

Other:

Is this a full powered effectiveness trial or a pilot/feasibility trial? *

Pilot/feasibility

Fully powered

Manuscript tracking number *

If this is a JMIR submission, please provide the manuscript tracking number under "other" (The ms tracking number can be found in the submission acknowledgement email, or when you login as author in JMIR. If the paper is already published in JMIR, then the ms tracking number is the four-digit number at the end of the DOI, to be found at the bottom of each published article in JMIR)

no ms number (yet) / not (yet) submitted to / published in JMIR

Other:

TITLE AND ABSTRACT

1a) TITLE: Identification as a randomized trial in the title

1a) Does your paper address CONSORT item 1a? *

I.e does the title contain the phrase "Randomized Controlled Trial"? (if not, explain the reason under "other")

yes

Other:

1a-i) Identify the mode of delivery in the title

Identify the mode of delivery. Preferably use “web-based” and/or “mobile” and/or “electronic game” in the title. Avoid ambiguous terms like “online”, “virtual”, “interactive”. Use “Internet-based” only if Intervention includes non-web-based Internet components (e.g. email), use “computer-based” or “electronic” only if offline products are used. Use “virtual” only in the context of “virtual reality” (3-D worlds). Use “online” only in the context of “online support groups”. Complement or substitute product names with broader terms for the class of products (such as “mobile” or “smart phone” instead of “iphone”), especially if the application runs on different platforms.

subitem not at all important

1

2

3

4

5

essential

Does your paper address subitem 1a-i? *

Copy and paste relevant sections from manuscript title (include quotes in quotation marks "like this" to indicate direct quotes from your manuscript), or elaborate on this item by providing additional information not in the ms, or briefly explain why the item is not applicable/relevant for your study

Double-blind, pragmatic, randomized placebo-controlled trial of 8-week self-administered at-home behavioral skills-based virtual reality (VR) for chronic low back pain

Note title includes: “randomized placebo-controlled trial”, “self-administered” and “Virtual Reality (VR)”

1a-ii) Non-web-based components or important co-interventions in title

Mention non-web-based components or important co-interventions in title, if any (e.g., “with telephone support”).

subitem not at all important

1

2

3

4

5

essential

Does your paper address subitem 1a-ii?

Copy and paste relevant sections from manuscript title (include quotes in quotation marks "like this" to indicate direct quotes from your manuscript), or elaborate on this item by providing additional information not in the ms, or briefly explain why the item is not applicable/relevant for your study

Your answer ???

1a-iii) Primary condition or target group in the title

Mention primary condition or target group in the title, if any (e.g., “for children with Type I Diabetes”) Example: A Web-based and Mobile Intervention with Telephone Support for Children with Type I Diabetes: Randomized Controlled Trial

subitem not at all important

1

2

3

4

5

essential

Does your paper address subitem 1a-iii? *

Copy and paste relevant sections from manuscript title (include quotes in quotation marks "like this" to indicate direct quotes from your manuscript), or elaborate on this item by providing additional information not in the ms, or briefly explain why the item is not applicable/relevant for your study

Double-blind, pragmatic, randomized placebo-controlled trial of 8-week self-administered at-home behavioral skills-based virtual reality (VR) for chronic low back pain

Note title includes: “chronic low back pain”

answer

1b) ABSTRACT: Structured summary of trial design, methods, results, and conclusions

NPT extension: Description of experimental treatment, comparator, care providers, centers, and blinding status.

1b-i) Key features/functionalities/components of the intervention and comparator in the METHODS section of the ABSTRACT

Mention key features/functionalities/components of the intervention and comparator in the abstract. If possible, also mention theories and principles used for designing the site. Keep in mind the needs of systematic reviewers and indexers by including important synonyms. (Note: Only report in the abstract what the main paper is reporting. If this information is missing from the main body of text, consider adding it)

subitem not at all important

1

2

3

4

5

essential

Does your paper address subitem 1b-i? *

Copy and paste relevant sections from the manuscript abstract (include quotes in quotation marks "like this" to indicate direct quotes from your manuscript), or elaborate on this item by providing additional information not in the ms, or briefly explain why the item is not applicable/relevant for your study

“Objective: To conduct a double-blind, parallel arm, single cohort, remote, randomized placebo-controlled trial for a self-administered behavioral skills-based VR program in community-based individuals with self-reported chronic low back pain during the COVID-19 pandemic.

Methods: A national online convenience sample of individuals with self-reported non-malignant low back pain ≥ 6 months duration and with average pain intensity ≥ 4/10 was enrolled and randomized 1:1 to one of two 56-day VR programs: (1) EaseVRx (pain relief skills immersive VR program); or (2) Sham VR (2D nature content delivered in a VR headset). Objective device use data and self-reported data were collected. The primary outcomes were change in average pain intensity and pain-related interference with activity, stress, mood, and sleep (baseline to end-of-treatment at day 56). Secondary outcomes were global impression of change and change in

physical function, sleep disturbance, pain self-efficacy, pain catastrophizing, pain acceptance, pain medication use, and user satisfaction. Analytic methods included intention-to-treat and a mixed-model framework."

1b-ii) Level of human involvement in the METHODS section of the ABSTRACT

Clarify the level of human involvement in the abstract, e.g., use phrases like "fully automated" vs. "therapist/nurse/care provider/physician-assisted" (mention number and expertise of providers involved, if any). (Note: Only report in the abstract what the main paper is reporting. If this information is missing from the main body of text, consider adding it)

subitem not at all important

1

2

3

4

5

essential

Does your paper address subitem 1b-ii?

Copy and paste relevant sections from the manuscript abstract (include quotes in quotation marks "like this" to indicate direct quotes from your manuscript), or elaborate on this item by providing additional information not in the ms, or briefly explain why the item is not applicable/relevant for your study

From our abstract: "To conduct a double-blind, parallel arm, single cohort, remote, randomized placebo-controlled trial for a self-administered behavioral skills-based VR program in community-based individuals with self-reported chronic low back pain during the COVID-19 pandemic. "

Note use of "Self-administered behavioral skills-based VR program"

1b-iii) Open vs. closed, web-based (self-assessment) vs. face-to-face assessments in the METHODS section of the ABSTRACT

Mention how participants were recruited (online vs. offline), e.g., from an open access website or from a clinic or a closed online user group (closed usergroup trial), and clarify if this was a purely web-based trial, or there were face-to-face components (as part of the intervention or for assessment). Clearly say if outcomes were self-assessed through questionnaires (as common in web-based trials). Note: In traditional offline trials, an open trial (open-label trial) is a type of clinical trial in which both the researchers and participants know which treatment is being administered. To avoid confusion, use “blinded” or “unblinded” to indicated the level of blinding instead of “open”, as “open” in web-based trials usually refers to “open access” (i.e. participants can self-enrol). (Note: Only report in the abstract what the main paper is reporting. If this information is missing from the main body of text, consider adding it)

subitem not at all important

1

2

3

4

5

essential

Does your paper address subitem 1b-iii?

Copy and paste relevant sections from the manuscript abstract (include quotes in quotation marks "like this" to indicate direct quotes from your manuscript), or elaborate on this item by providing additional information not in the ms, or briefly explain why the item is not applicable/relevant for your study

“Objective: To conduct a double-blind, parallel arm, single cohort, remote, randomized placebo-controlled trial for a self-administered behavioral skills-based VR program in community-based individuals with self-reported chronic low back pain during the COVID-19 pandemic.

Methods: A national online convenience sample of individuals with self-reported non-malignant low back pain > 6 months duration and with average pain intensity > 4/10 was enrolled and randomized 1:1 to one of two 56-day VR programs: (1) EaseVRx (pain relief skills immersive VR program); or (2) Sham VR (2D nature content delivered in a VR headset).”

Note following information in our abstract: double-blind, single cohort, remote, randomized placebo-controlled trial and online convenience sample

1b-iv) RESULTS section in abstract must contain use data

Report number of participants enrolled/assessed in each group, the use/uptake of the intervention (e.g., attrition/adherence metrics, use over time, number of logins etc.), in addition to primary/secondary outcomes. (Note: Only report in the abstract what the main paper is reporting. If this information is missing from the main body of text, consider adding it)

subitem not at all important

1

2

3

4

5

essential

Does your paper address subitem 1b-iv?

Copy and paste relevant sections from the manuscript abstract (include quotes in quotation marks "like this" to indicate direct quotes from your manuscript), or elaborate on this item by providing additional information not in the ms, or briefly explain why the item is not applicable/relevant for your study

Yes, from our abstract: “The study sample was 179 adults (female: 77%; Caucasian: 91%; at least some college education: 92%; mean age: 51.5 years, SD=13.1; average pain intensity: 5/10, SD=1.2; back pain duration >5 years: 67%). No group differences were found for any baseline variable or treatment engagement. User satisfaction ratings were higher for EaseVRx vs. Sham VR (p<0.0001). Both groups improved significantly for all five primary outcomes; EaseVRx was superior to Sham VR for all primary outcomes except pain-related sleep interference. For EaseVRx, large pre-post effect sizes ranged from 1.06-1.3 and met moderate to substantial clinical importance for reduced pain intensity and pain-related interference with activity, mood, and stress. A greater proportion of participants in the EaseVRx group achieved > 30% reduction in pain intensity (EaseVRx: 65.4%; Sham VR: 40.5%), and 46% of EaseVRx achieved >50% reduction in pain. Physical function and sleep disturbance significantly improved for both treatment groups with superior improvements found for EaseVRx (p=0.0019 and p=.0353, respectively). Pain catastrophizing, pain self-efficacy, prescription opioid use (morphine milligram equivalent; MME) did not reach statistical significance for either group. Use of over-the-counter analgesic use was reduced for EaseVRx (p<0.01) but not for ShamVR.“

1b-v) CONCLUSIONS/DISCUSSION in abstract for negative trials

Conclusions/Discussions in abstract for negative trials: Discuss the primary outcome - if the trial is negative (primary outcome not changed), and the intervention was not used, discuss whether negative results are attributable to lack of uptake and discuss reasons. (Note: Only report in the abstract what the main paper is reporting. If this information is missing from the main body of text, consider adding it)

subitem not at all important

1

2

3

4

5

essential

Does your paper address subitem 1b-v?

Copy and paste relevant sections from the manuscript abstract (include quotes in quotation marks "like this" to indicate direct quotes from your manuscript), or elaborate on this item by providing additional information not in the ms, or briefly explain why the item is not applicable/relevant for your study

Note this section from our abstract: “Conclusions: EaseVRx had high user satisfaction and superior and clinically meaningful symptom reduction for average pain intensity and pain-related interference with activity, mood, and stress compared to sham VR. Additional research is needed to determine durability of treatment effects and to characterize mechanisms of treatment effects. Home-based VR may expand access to effective and on-demand non-pharmacologic treatment for chronic low back pain.”

INTRODUCTION

2a) In INTRODUCTION: Scientific background and explanation of rationale

2a-i) Problem and the type of system/solution

Describe the problem and the type of system/solution that is object of the study: intended as stand-alone intervention vs. incorporated in broader health care program? Intended for a particular patient population? Goals of the

intervention, e.g., being more cost-effective to other interventions, replace or complement other solutions? (Note: Details about the intervention are provided in “Methods” under 5)

subitem not at all important

1

2

3

4

5

essential

Does your paper address subitem 2a-i? *

Copy and paste relevant sections from the manuscript (include quotes in quotation marks "like this" to indicate direct quotes from your manuscript), or elaborate on this item by providing additional information not in the ms, or briefly explain why the item is not applicable/relevant for your study

“The current study builds on this prior work and extends it in several ways. First, the VR treatment program being tested (EaseVRx) is 56 days in length thereby aligning more with traditional and reimbursable behavioral medicine programs such as eight-week chronic pain CBT or mindfulness programs. Second, the VR content was enriched with interoceptive entrainment techniques designed to enhance biofeedback response and learning. Third, the therapeutic VR program includes expanded pain neuroscience education, and it includes principles and elements drawn from CBT, mindfulness, and acceptance-based treatments for chronic pain. Fourth, the study includes a VR sham comparator to control for the novelty of the technology and placebo effects. Fifth, data for analgesic medication use was collected.“

2a-ii) Scientific background, rationale: What is known about the (type of) system

Scientific background, rationale: What is known about the (type of) system that is the object of the study (be sure to discuss the use of similar systems for other conditions/diagnoses, if appropiate), motivation for the study, i.e. what are the reasons for and what is the context for this specific study, from which stakeholder viewpoint is the study performed, potential impact of findings [2]. Briefly justify the choice of the comparator.

subitem not at all important

1

2

3

4

5

essential

Does your paper address subitem 2a-ii? *

Copy and paste relevant sections from the manuscript (include quotes in quotation marks "like this" to indicate direct quotes from your manuscript), or elaborate on this item by providing additional information not in the ms, or briefly explain why the item is not applicable/relevant for your study

“The scientific literature on VR for chronic pain includes studies conducted in complex regional pain syndrome, [35] chronic headache/migraine, [36] fibromyalgia, [37,38] and chronic musculoskeletal pain [39,40]. Two recent reviews and meta-analyses reported VR efficacy for reducing pain and disability (physical rehabilitation) for painful spinal conditions [41] and for orthopedic rehabilitation [42]. Such rehabilitation studies may apply interactive VR in isolation or with kinematic training [43]. In addition to small sample sizes, the literature for VR in chronic pain remains limited by studies conducted in experimental or clinical settings (vs. home-based and pragmatic studies), a lack of placebo controlled studies, and studies that have yielded low quality evidence [41]. Additionally, the literature has been largely restricted to VR content involving distraction or physical rehabilitation and kinematic exercises with little or no content on active behavioral pain management skills acquisition. To address several of these evidence gaps, our group recently conducted an RCT of a 21-day VR program that included chronic pain education and pain relief skills such as diaphragmatic breathing and relaxation response training, and cognition and emotion regulation techniques [17]. Individuals with CLBP or fibromyalgia were randomized to receive either the 21-day VR treatment program or the same treatment content delivered in audio-only format (N=74). At post-treatment, the VR skills-based treatment group evidenced superior reductions in pain intensity and pain-related interference with activity, sleep, mood, and stress compared to the audio treatment group, with results strengthening after two weeks. Similar treatment engagement rates between treatment groups supported a conclusion that the immersive effects of VR yielded superior outcomes.”

2b) In INTRODUCTION: Specific objectives or hypotheses

Does your paper address CONSORT subitem 2b? *

Copy and paste relevant sections from the manuscript (include quotes in quotation marks "like this" to indicate direct quotes from your manuscript), or elaborate on this item by providing additional information not in the ms, or briefly explain why the item is not applicable/relevant for your study

“Our objective was to conduct a placebo-controlled pragmatic RCT in community-based individuals with CLBP assigned to receive one of two 56-day treatment programs: therapeutic VR (EaseVRx) or Sham VR [44]. We hypothesized that participants assigned to therapeutic VR would evidence superior outcomes for all baseline to post-treatment comparisons compared to participants assigned to Sham VR.”

METHODS

3a) Description of trial design (such as parallel, factorial) including allocation ratio

Does your paper address CONSORT subitem 3a? *

Copy and paste relevant sections from the manuscript (include quotes in quotation marks "like this" to indicate direct quotes from your manuscript), or elaborate on this item by providing additional information not in the ms, or briefly explain why the item is not applicable/relevant for your study

“The study protocol is published elsewhere and provides additional detail.[44] We conducted a single-cohort, double-blinded (participant and analysts), cross-sectional, placebo-controlled randomized clinical trial in an online convenience national sample of community-based individuals with self-reported CLBP. Study participants were participating in a longer study of 8.5 months duration that involves multiple additional post-treatment assessments not reported here. The current report is constrained to the end of treatment time point (day 56). “

“Participants were randomized 1:1 and allocated to one of two treatment groups: (1) the 56-day pain relief skills VR program (EaseVRx) or (2) a 56-day control VR condition (Sham VR). REDCap Cloud (nPhase, Inc.; Encinitas, CA) was used to apply an automatic and blinded randomization program and ensure equal allocation to both groups. Participants and study statisticians were blinded to treatment group assignment. Statisticians performed blinded analysis of datasets that were randomly labeled Group A and Group B with statistician unblinding occurring only after post-treatment month 3 data were collected and the dataset

locked (post-treatment month 3 data not yet analyzed). The three study coordinators unblinded to individual treatment group assignments were not involved in any data analyses. Study participants remain blinded to treatment group assignment until their participation in the larger study is completed (8.5 months after randomization).”

3b) Important changes to methods after trial commencement (such as eligibility criteria), with reasons

Does your paper address CONSORT subitem 3b? *

Copy and paste relevant sections from the manuscript (include quotes in quotation marks "like this" to indicate direct quotes from your manuscript), or elaborate on this item by providing additional information not in the ms, or briefly explain why the item is not applicable/relevant for your study

Not applicable

3b-i) Bug fixes, Downtimes, Content Changes

Bug fixes, Downtimes, Content Changes: ehealth systems are often dynamic systems. A description of changes to methods therefore also includes important changes made on the intervention or comparator during the trial (e.g., major bug fixes or changes in the functionality or content) (5-iii) and other “unexpected events” that may have influenced study design such as staff changes, system failures/downtimes, etc. [2].

subitem not at all important

1

2

3

4

5

essential

Does your paper address subitem 3b-i?

Copy and paste relevant sections from the manuscript (include quotes in quotation marks "like this" to indicate direct quotes from your manuscript), or elaborate on this item by providing additional information not in the ms, or briefly explain why the item is not applicable/relevant for your study

Not applicable

4a) Eligibility criteria for participants

Does your paper address CONSORT subitem 4a? *

Copy and paste relevant sections from the manuscript (include quotes in quotation marks "like this" to indicate direct quotes from your manuscript), or elaborate on this item by providing additional information not in the ms, or briefly explain why the item is not applicable/relevant for your study

Yes, see: “Table 1. Study inclusion and exclusion criteria”.

4a-i) Computer / Internet literacy

Computer / Internet literacy is often an implicit “de facto” eligibility criterion - this should be explicitly clarified.

subitem not at all important

1

2

3

4

5

essential

Does your paper address subitem 4a-i?

Copy and paste relevant sections from the manuscript (include quotes in quotation marks "like this" to indicate direct quotes from your manuscript), or elaborate on this item by providing additional information not in the ms, or briefly explain why the item is not applicable/relevant for your study

From Table 1: “Implicit de facto internet and computer literacy”

4a-ii) Open vs. closed, web-based vs. face-to-face assessments:

Open vs. closed, web-based vs. face-to-face assessments: Mention how participants were recruited (online vs. offline), e.g., from an open access website or from a clinic, and clarify if this was a purely web-based trial, or there were face-to-face components (as part of the intervention or for assessment), i.e., to what degree got the study team to know the participant. In online-only trials, clarify if participants were quasi-anonymous and whether having multiple identities was possible or whether technical or logistical measures (e.g., cookies, email confirmation, phone calls) were used to detect/prevent these.

subitem not at all important

1

2

3

4

5

essential

Does your paper address subitem 4a-ii? *

Copy and paste relevant sections from the manuscript (include quotes in quotation marks "like this" to indicate direct quotes from your manuscript), or elaborate on this item by providing additional information not in the ms, or briefly explain why the item is not applicable/relevant for your study

“Community-based individuals with CLBP were recruited nationally through chronic pain organizations (e.g., American Chronic Pain Association) and through Facebook online advertisements. Additionally, study advertisements were emailed to professional contacts at several medical clinics with requests to forward amongst medical colleagues nationally. All study advertisements directed interested individuals with CLBP to the study website for information and invitation to complete an automated online eligibility form that screened for inclusion and exclusion criteria (see Table 1). This screening process was completed over the phone for two individuals due to technical difficulties. Individuals determined to be eligible for the study were invited to complete an electronic informed consent (eConsent; see Multimedia Appendix 1 for the study consent form) and provide their e-signature to complete their enrollment in the study. Following electronic informed consent.” and “All study procedures occurred remotely.”

4a-iii) Information giving during recruitment

Information given during recruitment. Specify how participants were briefed for recruitment and in the informed consent procedures (e.g., publish the informed consent documentation as appendix, see also item X26), as this information may have an effect on user self-selection, user expectation and may also bias results.

subitem not at all important

1

2

3

4

5

essential

Does your paper address subitem 4a-iii?

Copy and paste relevant sections from the manuscript (include quotes in quotation marks "like this" to indicate direct quotes from your manuscript), or elaborate on this item by providing additional information not in the ms, or briefly explain why the item is not applicable/relevant for your study

“Individuals determined to be eligible for the study were invited to participate in a study examining the effectiveness of an 8-week VR wellness program in helping them manage chronic lower back pain. If willing to participate, participants completed an electronic informed consent (eConsent; see Multimedia Appendix 1 for the study consent form) and provided their e-signature to complete their enrollment in the study. “

4b) Settings and locations where the data were collected

Does your paper address CONSORT subitem 4b? *

Copy and paste relevant sections from the manuscript (include quotes in quotation marks "like this" to indicate direct quotes from your manuscript), or elaborate on this item by providing additional information not in the ms, or briefly explain why the item is not applicable/relevant for your study

“Data collection included electronic participant-reported measures and objective VR device use data collected from the VR devices.”

4b-i) Report if outcomes were (self-)assessed through online questionnaires

Clearly report if outcomes were (self-)assessed through online questionnaires (as common in web-based trials) or otherwise.

subitem not at all important

1

2

3

4

5

essential

Does your paper address subitem 4b-i? *

Copy and paste relevant sections from the manuscript (include quotes in quotation marks "like this" to indicate direct quotes from your manuscript), or elaborate on this item by providing additional information not in the ms, or briefly explain why the item is not applicable/relevant for your study

“Data collection included electronic participant-reported measures and objective VR device use data collected from the VR devices.”

4b-ii) Report how institutional affiliations are displayed

Report how institutional affiliations are displayed to potential participants [on ehealth media], as affiliations with prestigious hospitals or universities may affect volunteer rates, use, and reactions with regards to an intervention.(Not a required item – describe only if this may bias results)

subitem not at all important

1

2

3

4

5

essential

Does your paper address subitem 4b-ii?

Copy and paste relevant sections from the manuscript (include quotes in quotation marks "like this" to indicate direct quotes from your manuscript), or elaborate on this item by providing additional information not in the ms, or briefly explain why the item is not applicable/relevant for your study

There were no institutional affiliations associated with this study.

5) The interventions for each group with sufficient details to allow replication, including how and when they were actually administered

5-i) Mention names, credential, affiliations of the developers, sponsors, and owners

Mention names, credential, affiliations of the developers, sponsors, and owners [6] (if authors/evaluators are owners or developer of the software, this needs to be declared in a “Conflict of interest” section or mentioned elsewhere in the manuscript).

subitem not at all important

1

2

3

4

5

essential

Does your paper address subitem 5-i?

Copy and paste relevant sections from the manuscript (include quotes in quotation marks "like this" to indicate direct quotes from your manuscript), or elaborate on this item by providing additional information not in the ms, or briefly explain why the item is not applicable/relevant for your study

Drs. Garcia and Maddox and Ian Mackey are employees of AppliedVR, Inc. Joshua Sackman is president of AppliedVR, Inc. Dr. Darnall is chief science advisor for AppliedVR, Inc. Drs. Birckhead, Krishnamurthy, and Salmasi are consultants for AppliedVR, Inc.

5-ii) Describe the history/development process

Describe the history/development process of the application and previous formative evaluations (e.g., focus groups, usability testing), as these will have an impact on adoption/use rates and help with interpreting results.

subitem not at all important

1

2

3

4

5

essential

Does your paper address subitem 5-ii?

Copy and paste relevant sections from the manuscript (include quotes in quotation marks "like this" to indicate direct quotes from your manuscript), or elaborate on this item by providing additional information not in the ms, or briefly explain why the item is not applicable/relevant for your study

This study was testing a Beta version of both the EaseVR and ShamVR Programs.

5-iii) Revisions and updating

Revisions and updating. Clearly mention the date and/or version number of the application/intervention (and comparator, if applicable) evaluated, or describe whether the intervention underwent major changes during the evaluation process, or whether the development and/or content was “frozen” during the trial. Describe dynamic components such as news feeds or changing content which may have an impact on the replicability of the intervention (for unexpected events see item 3b).

subitem not at all important

1

2

3

4

5

essential

Does your paper address subitem 5-iii?

Copy and paste relevant sections from the manuscript (include quotes in quotation marks "like this" to indicate direct quotes from your manuscript), or elaborate on this item by providing additional information not in the ms, or briefly explain why the item is not applicable/relevant for your study

This study was testing a Beta version of both the EaseVR and ShamVR Programs.

5-iv) Quality assurance methods

Provide information on quality assurance methods to ensure accuracy and quality of information provided [1], if applicable.

subitem not at all important

1

2

3

4

5

essential

Does your paper address subitem 5-iv?

Copy and paste relevant sections from the manuscript (include quotes in quotation marks "like this" to indicate direct quotes from your manuscript), or elaborate on this item by providing additional information not in the ms, or briefly explain why the item is not applicable/relevant for your study

Not applicable. Both programs tested (EaseVR and ShamVR) were uniformly standardized.

5-v) Ensure replicability by publishing the source code, and/or providing screenshots/screen-capture video, and/or providing flowcharts of the algorithms used

Ensure replicability by publishing the source code, and/or providing screenshots/screen-capture video, and/or providing flowcharts of the algorithms used. Replicability (i.e., other researchers should in principle be able to replicate the study) is a hallmark of scientific reporting.

subitem not at all important

1

2

3

4

5

essential

Does your paper address subitem 5-v?

Copy and paste relevant sections from the manuscript (include quotes in quotation marks "like this" to indicate direct quotes from your manuscript), or elaborate on this item by providing additional information not in the ms, or briefly explain why the item is not applicable/relevant for your study

The measures we used are open source. The software used was a beta version of a proprietary product that is commercially available.

5-vi) Digital preservation

Digital preservation: Provide the URL of the application, but as the intervention is likely to change or disappear over the course of the years; also make sure the intervention is archived (Internet Archive, webcitation.org, and/or

publishing the source code or screenshots/videos alongside the article). As pages behind login screens cannot be archived, consider creating demo pages which are accessible without login.

subitem not at all important

1

2

3

4

5

essential

Does your paper address subitem 5-vi?

Copy and paste relevant sections from the manuscript (include quotes in quotation marks "like this" to indicate direct quotes from your manuscript), or elaborate on this item by providing additional information not in the ms, or briefly explain why the item is not applicable/relevant for your study

This may be accessed through the owner, AppliedVR.

5-vii) Access

Access: Describe how participants accessed the application, in what setting/context, if they had to pay (or were paid) or not, whether they had to be a member of specific group. If known, describe how participants obtained “access to the platform and Internet” [1]. To ensure access for editors/reviewers/readers, consider to provide a “backdoor” login account or demo mode for reviewers/readers to explore the application (also important for archiving purposes, see vi).

subitem not at all important

1

2

3

4

5

essential

Does your paper address subitem 5-vii? *

Copy and paste relevant sections from the manuscript (include quotes in quotation marks "like this" to indicate direct quotes from your manuscript), or elaborate on this item by providing additional information not in the ms, or briefly explain why the item is not applicable/relevant for your study

“Participants in both treatment groups (EaseVRx and ShamVR) received a PICO G2 4K all-in-one head-mounted VR device at no cost through the mail.”

5-viii) Mode of delivery, features/functionalities/components of the intervention and comparator, and the theoretical framework

Describe mode of delivery, features/functionalities/components of the intervention and comparator, and the theoretical framework [6] used to design them (instructional strategy [1], behaviour change techniques, persuasive features, etc., see e.g., [7, 8] for terminology). This includes an in-depth description of the content (including where it is coming from and who developed it) [1],” whether [and how] it is tailored to individual circumstances and allows users to track their progress and receive feedback” [6]. This also includes a description of communication delivery channels and – if computer-mediated communication is a component – whether communication was synchronous or asynchronous [6]. It also includes information on presentation strategies [1], including page design principles, average amount of text on pages, presence of hyperlinks to other resources, etc. [1].

subitem not at all important

1

2

3

4

5

essential

Does your paper address subitem 5-viii? *

Copy and paste relevant sections from the manuscript (include quotes in quotation marks "like this" to indicate direct quotes from your manuscript), or elaborate on this item by providing additional information not in the ms, or briefly explain why the item is not applicable/relevant for your study

"Therapeutic VR (EaseVRx)

Participants randomized and allocated to this treatment group received an immersive multi-modal, skills-based, pain self-management VR program, called EaseVRx (AppliedVR; Los Angeles, CA), that incorporates evidence-based principles of CBT, mindfulness, and pain neuroscience education. The program content trains users on evidence-based pain and stress management strategies via immersive and enhanced biofeedback experiences. EaseVRx combines biopsychosocial education, diaphragmatic breathing training, relaxation response exercises that activate the parasympathetic nervous system, and executive functioning games to provide a mind-body approach toward living better with chronic pain. The standardized 56-day program delivers a multifaceted combination of pain relief skills training through a prescribed sequence of daily immersive experiences. Each VR experience is 2-16 minutes in length (average of 6 minutes). The VR treatment modules were designed to minimize triggers of emotional distress or cybersickness. Treatment module categories included:

- Pain education: visual and voice-guided lessons establish a medical and scientific rationale for the VR exercises and behavioral medicine skills for pain relief.
- Relaxation/Interoception: scenes that progressively change from busy/active to calm in order to train users to understand the benefits of progressive relaxation
- Mindful escapes: high-resolution 360 videos with therapeutic voiceovers, music, guided breathing and sound effects designed to maximize the relaxation response and participant engagement.
- Pain Distraction Games: interactive games to train the skill of shifting focus away from pain.
- Dynamic breathing: breathing-based biofeedback training in immersive and interactive environments to support self-regulation and relaxation. These modules become increasingly challenging as users increase their skill with diaphragmatic breathing and parasympathetic control.

Sham VR

In compliance with VR-CORE clinical trial guidelines, we selected an active control that utilizes non-immersive, two-dimensional (2D) content within a VR headset as the most rigorous VR placebo.[32] The Sham VR headset displayed 2D nature footage (e.g., wildlife in the savannah) with neutral music that was selected to be neither overly relaxing, aversive nor distracting. The experience of Sham VR is similar to viewing nature scenes on a large screen television and is not interactive. There were 20 videos that were rotated over the 56 sessions, with average duration of sessions closely matching those of EaseVRx.
"

5-ix) Describe use parameters

Describe use parameters (e.g., intended “doses” and optimal timing for use). Clarify what instructions or recommendations were given to the user, e.g., regarding timing, frequency, heaviness of use, if any, or was the intervention used ad libitum.

subitem not at all important

1

2

3

4

5

essential

Does your paper address subitem 5-ix?

Copy and paste relevant sections from the manuscript (include quotes in quotation marks "like this" to indicate direct quotes from your manuscript), or elaborate on this item by providing additional information not in the ms, or briefly explain why the item is not applicable/relevant for your study

“Participants in both groups were instructed to complete one VR program session daily for 56 days. “

5-x) Clarify the level of human involvement

Clarify the level of human involvement (care providers or health professionals, also technical assistance) in the e-intervention or as co-intervention (detail number and expertise of professionals involved, if any, as well as “type of assistance offered, the timing and frequency of the support, how it is initiated, and the medium by which the assistance is delivered”. It may be necessary to distinguish between the level of human involvement required for the trial, and the level of human involvement required for a routine application outside of a RCT setting (discuss under item 21 – generalizability).

subitem not at all important

1

2

3

4

5

essential

Does your paper address subitem 5-x?

Copy and paste relevant sections from the manuscript (include quotes in quotation marks "like this" to indicate direct quotes from your manuscript), or elaborate on this item by providing additional information not in the ms, or briefly explain why the item is not applicable/relevant for your study

Human involvement was restricted to enrollment screening, receipt of VR device, and any participant outreach to study staff.

“ Study staff monitored participant completion of the twice-weekly surveys and device use. Study staff provided reminders to complete surveys and otherwise were available upon request for technical support. “

5-xi) Report any prompts/reminders used

Report any prompts/reminders used: Clarify if there were prompts (letters, emails, phone calls, SMS) to use the application, what triggered them, frequency etc. It may be necessary to distinguish between the level of prompts/reminders required for the trial, and the level of prompts/reminders for a routine application outside of a RCT setting (discuss under item 21 – generalizability).

subitem not at all important

1

2

3

4

5

essential

Does your paper address subitem 5-xi? *

Copy and paste relevant sections from the manuscript (include quotes in quotation marks "like this" to indicate direct quotes from your manuscript), or elaborate on this item by providing additional information not in the ms, or briefly explain why the item is not applicable/relevant for your study

Participants received SMS prompts to complete study surveys at designated time points.

5-xii) Describe any co-interventions (incl. training/support)

Describe any co-interventions (incl. training/support): Clearly state any interventions that are provided in addition to the targeted eHealth intervention, as ehealth intervention may not be designed as stand-alone intervention. This includes training sessions and support [1]. It may be necessary to distinguish between the level of training required for the trial, and the level of training for a routine application outside of a RCT setting (discuss under item 21 – generalizability.

subitem not at all important

1

2

3

4

5

essential

Does your paper address subitem 5-xii? *

Copy and paste relevant sections from the manuscript (include quotes in quotation marks "like this" to indicate direct quotes from your manuscript), or elaborate on this item by providing additional information not in the ms, or briefly explain why the item is not applicable/relevant for your study

Participants in this study were provided with online access to instructional materials outlining general use and set-up of the headset.

6a) Completely defined pre-specified primary and secondary outcome measures, including how and when they were assessed

Does your paper address CONSORT subitem 6a? *

Copy and paste relevant sections from the manuscript (include quotes in quotation marks "like this" to indicate direct quotes from your manuscript), or elaborate on this item by providing additional information not in the ms, or briefly explain why the item is not applicable/relevant for your study

“Data collection included electronic participant-reported measures and objective VR device use data collected from the VR devices.

Data were collected across four phases of the study: baseline, pre-treatment (days -14 to 0), active treatment (days 1-55), and end of treatment (day 56). The 14-day pre-treatment phase involved administering one survey three times; these survey scores were averaged within subjects to establish a single pre-treatment score for each variable assessed. During the eight-week active treatment phase, surveys were distributed biweekly (16 total surveys during treatment) and at end of treatment on day 56. Accordingly, there were 19 time points per participant.
“

6a-i) Online questionnaires: describe if they were validated for online use and apply CHERRIES items to describe how the questionnaires were designed/deployed

If outcomes were obtained through online questionnaires, describe if they were validated for online use and apply CHERRIES items to describe how the questionnaires were designed/deployed [9].

subitem not at all important

1

2

3

4

5

essential

Does your paper address subitem 6a-i?

Copy and paste relevant sections from manuscript text

All measures used are validated and used in online research.

6a-ii) Describe whether and how “use” (including intensity of use/dosage) was defined/measured/monitored

Describe whether and how “use” (including intensity of use/dosage) was defined/measured/monitored (logins, logfile analysis, etc.). Use/adoption metrics are important process outcomes that should be reported in any ehealth trial.

subitem not at all important

1

2

3

4

5

essential

Does your paper address subitem 6a-ii?

Copy and paste relevant sections from manuscript text

Use was defined as the data recorded by the devices (date and time stamped for device access and duration of use).

6a-iii) Describe whether, how, and when qualitative feedback from participants was obtained

Describe whether, how, and when qualitative feedback from participants was obtained (e.g., through emails, feedback forms, interviews, focus groups).

subitem not at all important

1

2

3

4

5

essential

Does your paper address subitem 6a-iii?

Copy and paste relevant sections from manuscript text

Not applicable

6b) Any changes to trial outcomes after the trial commenced, with reasons

Does your paper address CONSORT subitem 6b? *

Copy and paste relevant sections from the manuscript (include quotes in quotation marks "like this" to indicate direct quotes from your manuscript), or elaborate on this item by providing additional information not in the ms, or briefly explain why the item is not applicable/relevant for your study

Due to an error with the electronic survey administration satisfaction with treatment data was captured at one month post-treatment rather than at the 56-day assessment.

7a) How sample size was determined

NPT: When applicable, details of whether and how the clustering by care provides or centers was addressed

7a-i) Describe whether and how expected attrition was taken into account when calculating the sample size

Describe whether and how expected attrition was taken into account when calculating the sample size.

subitem not at all important

1

2

3

4

5

essential

Does your paper address subitem 7a-i?

Copy and paste relevant sections from manuscript title (include quotes in quotation marks "like this" to indicate direct quotes from your manuscript), or elaborate on this item by providing additional information not in the ms, or briefly explain why the item is not applicable/relevant for your study

“A power analysis was performed using data from a prior RCT of a 21-day at-home VR for chronic pain compared to an audio-only version of the treatment.[17] Results for this study revealed an average pre-post treatment difference score in pain intensity was 1.48 for the VR group and .756 for the audio-only group (on an 11-point scale). Assuming an alpha level of .05 and 90% power, 45 participants per group would detect a treatment group by time interaction. To buffer against potential high attrition (40%), a minimum of 75 participants would be required per treatment group with 90 participants per treatment group being ideal. ”

7b) When applicable, explanation of any interim analyses and stopping guidelines

Does your paper address CONSORT subitem 7b? *

Copy and paste relevant sections from the manuscript (include quotes in quotation marks "like this" to indicate direct quotes from your manuscript), or elaborate on this item by providing additional information not in the ms, or briefly explain why the item is not applicable/relevant for your study

Not applicable

8a) Method used to generate the random allocation sequence

NPT: When applicable, how care providers were allocated to each trial group

Does your paper address CONSORT subitem 8a? *

Copy and paste relevant sections from the manuscript (include quotes in quotation marks "like this" to indicate direct quotes from your manuscript), or elaborate on this item by providing additional information not in the ms, or briefly explain why the item is not applicable/relevant for your study

“Participants were randomized 1:1 and allocated to one of two treatment groups: (1) the 56-day pain relief skills VR program (EaseVRx) or (2) a 56-day control VR condition (Sham VR).

REDCap Cloud (nPhase, Inc.; Encinitas, CA) was used to apply an automatic and blinded randomization program and ensure equal allocation to both groups."

8b) Type of randomisation; details of any restriction (such as blocking and block size)

Does your paper address CONSORT subitem 8b? *

Copy and paste relevant sections from the manuscript (include quotes in quotation marks "like this" to indicate direct quotes from your manuscript), or elaborate on this item by providing additional information not in the ms, or briefly explain why the item is not applicable/relevant for your study

Not applicable

9) Mechanism used to implement the random allocation sequence (such as sequentially numbered containers), describing any steps taken to conceal the sequence until interventions were assigned

Does your paper address CONSORT subitem 9? *

Copy and paste relevant sections from the manuscript (include quotes in quotation marks "like this" to indicate direct quotes from your manuscript), or elaborate on this item by providing additional information not in the ms, or briefly explain why the item is not applicable/relevant for your study

Not applicable

10) Who generated the random allocation sequence, who enrolled participants, and who assigned participants to interventions

Does your paper address CONSORT subitem 10? *

Copy and paste relevant sections from the manuscript (include quotes in quotation marks "like this" to indicate direct quotes from your manuscript), or elaborate on this item by providing additional information not in the ms, or briefly explain why the item is not applicable/relevant for your study

Participants were randomized 1:1 and allocated via RedCap Cloud. Study coordinators enrolled and monitored progress.

11a) If done, who was blinded after assignment to interventions (for example, participants, care providers, those assessing outcomes) and how

NPT: Whether or not administering co-interventions were blinded to group assignment

11a-i) Specify who was blinded, and who wasn’t

Specify who was blinded, and who wasn’t. Usually, in web-based trials it is not possible to blind the participants [1, 3] (this should be clearly acknowledged), but it may be possible to blind outcome assessors, those doing data analysis or those administering co-interventions (if any).

subitem not at all important

1

2

3

4

5

essential

Does your paper address subitem 11a-i? *

Copy and paste relevant sections from the manuscript (include quotes in quotation marks "like this" to indicate direct quotes from your manuscript), or elaborate on this item by providing additional information not in the ms, or briefly explain why the item is not applicable/relevant for your study

“Participants and study statisticians were blinded to treatment group assignment. Statisticians performed blinded analysis of datasets that were randomly labeled Group A and Group B with statistician unblinding occurring only after post-treatment month 3 data were collected and the dataset locked (post-treatment month 3 data not yet analyzed). The three study coordinators unblinded to individual treatment group assignments were not involved in any data analyses. Study participants remain blinded to treatment group assignment until their participation in the larger study is completed (8.5 months after randomization).”

11a-ii) Discuss e.g., whether participants knew which intervention was the “intervention of interest” and which one was the “comparator”

Informed consent procedures (4a-ii) can create biases and certain expectations - discuss e.g., whether participants knew which intervention was the “intervention of interest” and which one was the “comparator”.

subitem not at all important

1

2

3

4

5

essential

Does your paper address subitem 11a-ii?

Copy and paste relevant sections from the manuscript (include quotes in quotation marks "like this" to indicate direct quotes from your manuscript), or elaborate on this item by providing additional information not in the ms, or briefly explain why the item is not applicable/relevant for your study

“Participants and study statisticians were blinded to treatment group assignment.”

11b) If relevant, description of the similarity of interventions

(this item is usually not relevant for ehealth trials as it refers to similarity of a placebo or sham intervention to a active medication/intervention)

Does your paper address CONSORT subitem 11b? *

Copy and paste relevant sections from the manuscript (include quotes in quotation marks "like this" to indicate direct quotes from your manuscript), or elaborate on this item by providing additional information not in the ms, or briefly explain why the item is not applicable/relevant for your study

“Participants in both treatment groups (EaseVRx and ShamVR) received a PICO G2 4K all-in-one head-mounted VR device at no cost through the mail. The PICO G2 4K device was used because they are commercially available, widely used, inexpensive, have minimal visual latency, and are easier for participants to use than many other devices. This hardware allows for displaying 3D images (EaseVRx) and 2D images (Sham VR). While each VR device contained software specific to the individual participant’s assigned VR treatment group, all device packaging, and directions for use were common to both treatment groups. Participants in this study were provided with online access to instructional materials outlining general use and set-up of the headset. Relevant to the EaseVRx group, user exhalation is measured by the

microphone embedded in the Pico G2 hardware, offering biodata-enabled immersive therapeutics. Participants in both groups were instructed to complete one VR program session daily for 56 days. Study staff monitored participant completion of the twice-weekly surveys and device use. Study staff provided reminders to complete surveys and otherwise were available upon request for technical support.“

12a) Statistical methods used to compare groups for primary and secondary outcomes

NPT: When applicable, details of whether and how the clustering by care providers or centers was addressed

Does your paper address CONSORT subitem 12a? *

Copy and paste relevant sections from the manuscript (include quotes in quotation marks "like this" to indicate direct quotes from your manuscript), or elaborate on this item by providing additional information not in the ms, or briefly explain why the item is not applicable/relevant for your study

“All analyses involved two-sided hypothesis tests, with α=0.05 and adjusted for any multiple comparisons within the family of tests as appropriate. Group equivalence was assessed through univariate tests of association between treatment groups (EaseVRx/Sham VR) for all baseline demographic and clinical variables with Chi-Square and Kruskal-Wallis applied as appropriate.

The data were analyzed in a mixed-model framework (PROC GLIMMIX in SAS®) using a marginal (population-averaged) model to allow for correlated responses across the repeated measures. There were three explanatory factors; treatment group, time and time × treatment group. Treatment group, EaseVRx vs. Sham VR was specified as a fixed-effects factor. Time was specified as a random-effects factor to allow for correlated response using heterogeneous compound symmetry for the covariance structure within time. The effect of interest is the time × treatment group effect which tests whether the treatment group influenced the trajectory of the key variables over time.

Data were 95.05% complete; missing values were not imputed for estimation of effects but the predicted means were used in the graphical description. The primary outcomes were the time course of DVPRS (pain scale) from baseline (defined as the average of three pain ratings obtained during the two weeks before enrollment/randomization), at eight weekly time points (twice per week) across the 8-week treatment period, and immediately post-treatment (Day 56). A linear mixed model was used with the treatment group (EaseVRx vs. Sham VR) as an independent-groups factor (i.e., a between-subjects factor) and time of measurement as a dependent groups factor (i.e., a within-subjects factor). DVPRS-II measures were analyzed using the same approach. Effect sizes (baseline to immediately post-treatment completion at day 56) were computed by treatment group using an adaptation of Cohen *d* to suit the repeated measures design. [70] ”

12a-i) Imputation techniques to deal with attrition / missing values

Imputation techniques to deal with attrition / missing values: Not all participants will use the intervention/comparator as intended and attrition is typically high in ehealth trials. Specify how participants who did not use the application or dropped out from the trial were treated in the statistical analysis (a complete case analysis is strongly discouraged, and simple imputation techniques such as LOCF may also be problematic [4]).

subitem not at all important

1

2

3

4

5

essential

Does your paper address subitem 12a-i? *

Copy and paste relevant sections from the manuscript (include quotes in quotation marks "like this" to indicate direct quotes from your manuscript), or elaborate on this item by providing additional information not in the ms, or briefly explain why the item is not applicable/relevant for your study

“Data were 95.05% complete; missing values were not imputed for estimation of effects but the predicted means were used in the graphical description. “

12b) Methods for additional analyses, such as subgroup analyses and adjusted analyses

Does your paper address CONSORT subitem 12b? *

Copy and paste relevant sections from the manuscript (include quotes in quotation marks "like this" to indicate direct quotes from your manuscript), or elaborate on this item by providing additional information not in the ms, or briefly explain why the item is not applicable/relevant for your study

None

X26) REB/IRB Approval and Ethical Considerations [recommended as subheading under "Methods"] (not a CONSORT item)

X26-i) Comment on ethics committee approval

subitem not at all important

1

2

3

4

5

essential

Does your paper address subitem X26-i?

Copy and paste relevant sections from the manuscript (include quotes in quotation marks "like this" to indicate direct quotes from your manuscript), or elaborate on this item by providing additional information not in the ms, or briefly explain why the item is not applicable/relevant for your study

“The study was approved by IRB on July 2nd, 2020 and data collection for this report was completed in November 2020.”

x26-ii) Outline informed consent procedures

Outline informed consent procedures e.g., if consent was obtained offline or online (how? Checkbox, etc.?), and what information was provided (see 4a-ii). See [6] for some items to be included in informed consent documents.

subitem not at all important

1

2

3

4

5

essential

Does your paper address subitem X26-ii?

Copy and paste relevant sections from the manuscript (include quotes in quotation marks "like this" to indicate direct quotes from your manuscript), or elaborate on this item by providing additional information not in the ms, or briefly explain why the item is not applicable/relevant for your study

“If willing to participate, participants completed an electronic informed consent (eConsent; see Multimedia Appendix 1 for the study consent form) and provided their e-signature to complete their enrollment in the study.”

X26-iii) Safety and security procedures

Safety and security procedures, incl. privacy considerations, and any steps taken to reduce the likelihood or detection of harm (e.g., education and training, availability of a hotline)

subitem not at all important

1

2

3

4

5

essential

Does your paper address subitem X26-iii?

Copy and paste relevant sections from the manuscript (include quotes in quotation marks "like this" to indicate direct quotes from your manuscript), or elaborate on this item by providing additional information not in the ms, or briefly explain why the item is not applicable/relevant for your study

The consent form (appendix) offers the following details from the consent form:

“**RISKS AND DISCOMFORTS**

Virtual Reality Intervention

Participants using the virtual reality intervention may experience side effects common to users of VR and individuals who view 3D videos, including motion sickness, dizziness, eye strain, headaches, or other visual abnormalities. If participants experience these symptoms while using the device at home, they should stop using the device for 15 minutes and then attempt to resume the use of the device. If the symptoms do not resolve or recur and you are unable to continue the use of VR, you should contact the study team at the phone number listed on the first page. A small number of patients (up to 0.025%) may experience seizures or severe symptoms (e.g., disorientation, nausea, or drowsiness) upon viewing the virtual reality experience. Seizures from flashing light are more common in children and epileptic patients (who are excluded). To minimize this concern further, we have *not* incorporated flashing lights into the VR experiences. Some patients may find the VR goggles uncomfortable to wear or confining. To date, patients with claustrophobia have *not reported* discomfort using VR goggles. Nevertheless, individuals previously diagnosed with claustrophobia should discontinue use if they feel uncomfortable.

Questionnaires:
Some of the items in the surveys may make you feel uncomfortable or embarrassed. You are not required to respond to any item that you do not wish to answer. The surveys will be labeled with a unique study number that will link your identity so that only the research team can recognize you.

There are no anticipated long-term risks from participating in this study. There is the possible risk of loss of confidentiality of your research information. There may be unknown risks.“

RESULTS

13a) For each group, the numbers of participants who were randomly assigned, received intended treatment, and were analysed for the primary outcome

NPT: The number of care providers or centers performing the intervention in each group and the number of patients treated by each care provider in each center

Does your paper address CONSORT subitem 13a? *

“A total of 179 individuals received a VR device with their assigned treatment (EaseVRx n=89; Sham VR n=90).”

13b) For each group, losses and exclusions after randomisation, together with reasons

Does your paper address CONSORT subitem 13b? (NOTE: Preferably, this is shown in a CONSORT flow diagram) *

“Because intention-to-treat analyses were performed, the analytic dataset includes 11 individuals who did not provide complete data”

13b-i) Attrition diagram

Strongly recommended: An attrition diagram (e.g., proportion of participants still logging in or using the intervention/comparator in each group plotted over time, similar to a survival curve) or other figures or tables demonstrating usage/dose/engagement.

subitem not at all important

1

2

3

4

5

essential

Does your paper address subitem 13b-i?

Copy and paste relevant sections from the manuscript or cite the figure number if applicable (include quotes in quotation marks "like this" to indicate direct quotes from your manuscript), or elaborate on this item by providing additional information not in the ms, or briefly explain why the item is not applicable/relevant for your study

We provide this information in our consort diagram.

14a) Dates defining the periods of recruitment and follow-up

Does your paper address CONSORT subitem 14a? *

Copy and paste relevant sections from the manuscript (include quotes in quotation marks "like this" to indicate direct quotes from your manuscript), or elaborate on this item by providing additional information not in the ms, or briefly explain why the item is not applicable/relevant for your study

“The study was approved by IRB on July 2nd, 2020 and data collection for this report was completed in November 2020.”

14a-i) Indicate if critical “secular events” fell into the study period

Indicate if critical “secular events” fell into the study period, e.g., significant changes in Internet resources available or “changes in computer hardware or Internet delivery resources”

subitem not at all important

1

2

3

4

5

essential

Does your paper address subitem 14a-i?

Copy and paste relevant sections from the manuscript (include quotes in quotation marks "like this" to indicate direct quotes from your manuscript), or elaborate on this item by providing additional information not in the ms, or briefly explain why the item is not applicable/relevant for your study

Not applicable

14b) Why the trial ended or was stopped (early)

Does your paper address CONSORT subitem 14b? *

Copy and paste relevant sections from the manuscript (include quotes in quotation marks "like this" to indicate direct quotes from your manuscript), or elaborate on this item by providing additional information not in the ms, or briefly explain why the item is not applicable/relevant for your study

Not applicable

15) A table showing baseline demographic and clinical characteristics for each group

NPT: When applicable, a description of care providers (case volume, qualification, expertise, etc.) and centers (volume) in each group

Does your paper address CONSORT subitem 15? *

Copy and paste relevant sections from the manuscript (include quotes in quotation marks "like this" to indicate direct quotes from your manuscript), or elaborate on this item by providing additional information not in the ms, or briefly explain why the item is not applicable/relevant for your study

Yes, this information is present on Table 3. **Baseline Demographic Characteristics by Treatment Group**

The formatting does not paste well but here it is:

Table 3. Baseline Demographic Characteristics by Treatment Group

	**Treatment Group**		
	**EaseVRx (N=89)**	**Sham VR (N=90)**	**P-value**
**Gender**, n (%)			0.527[1]
Male	22 (25)	19 (21)	
Female	67 (75)	70 (78)	
Other	0 (0)	1 (1)	
**Age** (years)			0.964[2]
Mean (SD)	51.5 (13.5)	51.4 (12.9)	
Range	18.0, 81.0	25.0, 81.0	
Median (IQR)	51.0 (40.0, 62.0)	54.0 (41.0, 62.0)	
**Race**, n (%)			0.247[1]
Asian	2 (2)	1 (1)	
Caucasian	78 (88)	84 (94)	
African American	5 (6)	1 (1)	
Multi-Racial	2 (2)	3 (3)	
Other	2 (2)	0 (0)	
Missing	0	1	
**Education**, n (%)			0.142[1]
High School Graduate	6 (7)	9 (10)	
Some College	21 (24)	17 (19)	
Associate	10 (11)	16 (18)	
Undergraduate	17 (19)	25 (28)	
Post-Graduate	35 (39)	22 (25)	
Missing	0	1	
**Employment Status**, n (%)			0.781[1]
Part Time	9 (10)	7 (8)	
Full Time	37 (42)	34 (38)	
Not Working	13 (15)	10 (11)	
Retired	15 (17)	20 (22)	
Unable to Work	15 (17)	18 (20)	
Missing	0	1	
**Annual Household Income**, n (%)			0.665[1]

< $40,000	22 (25)	22 (24)	
$40,000 to $59,999	24 (27)	18 (20)	
$60,000 to $79,999	16 (18)	18 (20)	
≥ $80,000	26 (30)	32 (36)	
Missing	1	0	
**Marital Status**, n (%)			0.605[1]
Married/Civil Union	52 (59)	61 (68)	
Divorced/Widowed/Separated	20 (23)	14 (16)	
Single	10 (11)	10 (11)	
Cohabitating	6 (7)	5 (6)	
Missing	1	0	

[1]Chi-Square p-value; [2]Kruskal-Wallis p-value

15-i) Report demographics associated with digital divide issues

In ehealth trials it is particularly important to report demographics associated with digital divide issues, such as age, education, gender, social-economic status, computer/Internet/ehealth literacy of the participants, if known.

subitem not at all important

1

2

3

4

5

essential

Does your paper address subitem 15-i? *

Copy and paste relevant sections from the manuscript (include quotes in quotation marks "like this" to indicate direct quotes from your manuscript), or elaborate on this item by providing additional information not in the ms, or briefly explain why the item is not applicable/relevant for your study

None of the demographic data were predictive.

16) For each group, number of participants (denominator) included in each analysis and whether the analysis was by original assigned groups

16-i) Report multiple “denominators” and provide definitions

Report multiple “denominators” and provide definitions: Report N’s (and effect sizes) “across a range of study participation [and use] thresholds” [1], e.g., N exposed, N consented, N used more than x times, N used more than y weeks, N participants “used” the intervention/comparator at specific pre-defined time points of interest (in absolute and relative numbers per group). Always clearly define “use” of the intervention.

subitem not at all important

1

2

3

4

5

essential

Does your paper address subitem 16-i? *

Copy and paste relevant sections from the manuscript (include quotes in quotation marks "like this" to indicate direct quotes from your manuscript), or elaborate on this item by providing additional information not in the ms, or briefly explain why the item is not applicable/relevant for your study

“A total of 188 individuals were enrolled, randomized and allocated to the treatment group. After randomization, 9 individuals discontinued participation; (n=3) were unable to receive a VR device, (n=1) returned their unopened device due to a recent medical diagnosis, and (n=5) voluntarily withdrew for unknown reasons. A total of 179 individuals received a VR device with their assigned treatment (EaseVRx n=89; Sham VR n=90). Because intention-to-treat analyses were performed, the analytic dataset includes 11 individuals who did not provide complete data. A total of 168 participants (91%) completed the Day 56 assessment (end of treatment).”

“Effect sizes (baseline to immediately post-treatment completion at day 56) were computed by treatment group using an adaptation of Cohen *d* to suit the repeated measures design.[70]”

16-ii) Primary analysis should be intent-to-treat

Primary analysis should be intent-to-treat, secondary analyses could include comparing only “users”, with the appropriate caveats that this is no longer a randomized sample (see 18-i).

subitem not at all important

1

2

3

4

5

essential

Does your paper address subitem 16-ii?

Copy and paste relevant sections from the manuscript (include quotes in quotation marks "like this" to indicate direct quotes from your manuscript), or elaborate on this item by providing additional information not in the ms, or briefly explain why the item is not applicable/relevant for your study

“A total of 179 individuals received a VR device with their assigned treatment (EaseVRx n=89; Sham VR n=90). Because intention-to-treat analyses were performed, the analytic dataset includes 11 individuals who did not provide complete data. A total of 168 participants (91%) completed the Day 56 assessment (end of treatment).”

17a) For each primary and secondary outcome, results for each group, and the estimated effect size and its precision (such as 95% confidence interval)

Does your paper address CONSORT subitem 17a? *

Copy and paste relevant sections from the manuscript (include quotes in quotation marks "like this" to indicate direct quotes from your manuscript), or elaborate on this item by providing additional information not in the ms, or briefly explain why the item is not applicable/relevant for your study

The confidence intervals are present in the figures for each below.

“Pain intensity reduced by an average of 41.6% for EaseVRx and 23.5% for the Sham VR. Cohen’s *d* for the EaseVRx was 1.3, with combined results showing large effect size and moderate clinical importance. The VR Sham group effect size was 0.84, with combined results showing a large effect size and minimal clinical importance.”

“Pain-Interference with Activity reduced by an average of 49.6% for EaseVRx and 30.7% for the Sham VR. Cohen’s *d* for the EaseVRx was 1.19, with combined results showing large effect

size and moderate clinical importance. Seventy-three percent (n=61) of EaseVRx and 51%(n=43) of Sham VR participants achieved ≥30% reduction in Pain-Related Interference with Activity, and 51 (n=43) achieved ≥50% reduction. The VR Sham group effect size was 0.96, with combined results showing a large effect size and moderate clinical importance."

"Pain-Interference with Mood reduced by an average of 49.9% for EaseVRx and 41.8% for the Sham VR. Cohen's *d* for the EaseVRx was 1.06, with combined results evidencing a large effect size and substantial clinical importance. Seventy-one percent (n=60) of EaseVRx participants achieved ≥30% reduction in Pain-Related Interference with Mood, and 63.1% (n=53) achieved ≥50% reduction. The VR Sham group effect size was 0.79, with combined results showing a moderate effect size and moderate clinical importance."

"Pain-Interference with Sleep reduced by an average of 54% for EaseVRx and 29.4% for the Sham VR. Cohen's *d* for the EaseVRx was 0.95, with combined results showing large effect size and substantial clinical importance for symptom reduction. Sixty-nine percent (n=58) of EaseVRx participants achieved ≥30% reduction in Pain-Related Interference with Mood, and 60% (n=50) achieved ≥50% reduction. The VR Sham group effect size was 0.88, with combined results showing a large effect size and moderate clinical importance."

"Pain-Interference with Stress reduced by an average of 58.1% for EaseVRx and 39.2% for the Sham VR. Cohen's *d* for the EaseVRx was 1.18, with combined results evidencing a large effect size and substantial clinical importance. Seventy-six percent (n=64) of EaseVRx participants achieved ≥30% reduction in Pain-Related Interference with Stress, and 68% (n=57) achieved ≥50% reduction. The VR Sham group effect size was 0.84, with combined results showing a large effect size and moderate clinical importance."

17a-i) Presentation of process outcomes such as metrics of use and intensity of use

In addition to primary/secondary (clinical) outcomes, the presentation of process outcomes such as metrics of use and intensity of use (dose, exposure) and their operational definitions is critical. This does not only refer to metrics of attrition (13-b) (often a binary variable), but also to more continuous exposure metrics such as "average session length". These must be accompanied by a technical description how a metric like a "session" is defined (e.g., timeout after idle time) [1] (report under item 6a).

subitem not at all important

1

2

3

4

5

essential

Does your paper address subitem 17a-i?

Copy and paste relevant sections from the manuscript (include quotes in quotation marks "like this" to indicate direct quotes from your manuscript), or elaborate on this item by providing additional information not in the ms, or briefly explain why the item is not applicable/relevant for your study

“Treatment Engagement. Device use data were received for 149 participants (EaseVRx =77; ShamVR = 72). EaseVRx participants completed a mean of 43.3 sessions while the ShamVR group completed 48.1 sessions. A significant group difference for treatment engagement was not found.”

r17b) For binary outcomes, presentation of both absolute and relative effect sizes is recommended

Does your paper address CONSORT subitem 17b? *

Copy and paste relevant sections from the manuscript (include quotes in quotation marks "like this" to indicate direct quotes from your manuscript), or elaborate on this item by providing additional information not in the ms, or briefly explain why the item is not applicable/relevant for your study

“Pain intensity reduced by an average of 41.6% for EaseVRx and 23.5% for the Sham VR. Cohen’s *d* for the EaseVRx was 1.3, with combined results showing large effect size and moderate clinical importance. The VR Sham group effect size was 0.84, with combined results showing a large effect size and minimal clinical importance.”

“Pain-Interference with Activity reduced by an average of 49.6% for EaseVRx and 30.7% for the Sham VR. Cohen’s *d* for the EaseVRx was 1.19, with combined results showing large effect size and moderate clinical importance. Seventy-three percent (n=61) of EaseVRx and 51%(n=43) of Sham VR participants achieved ≥30% reduction in Pain-Related Interference with Activity, and 51 (n=43) achieved ≥50% reduction. The VR Sham group effect size was 0.96, with combined results showing a large effect size and moderate clinical importance.”

“Pain-Interference with Mood reduced by an average of 49.9% for EaseVRx and 41.8% for the Sham VR. Cohen’s *d* for the EaseVRx was 1.06, with combined results evidencing a large effect size and substantial clinical importance. Seventy-one percent (n=60) of EaseVRx participants achieved ≥30% reduction in Pain-Related Interference with Mood, and 63.1% (n=53) achieved

≥50% reduction. The VR Sham group effect size was 0.79, with combined results showing a moderate effect size and moderate clinical importance."

"Pain-Interference with Sleep reduced by an average of 54% for EaseVRx and 29.4% for the Sham VR. Cohen's *d* for the EaseVRx was 0.95, with combined results showing large effect size and substantial clinical importance for symptom reduction. Sixty-nine percent (n=58) of EaseVRx participants achieved ≥30% reduction in Pain-Related Interference with Mood, and 60% (n=50) achieved ≥50% reduction. The VR Sham group effect size was 0.88, with combined results showing a large effect size and moderate clinical importance."

"Pain-Interference with Stress reduced by an average of 58.1% for EaseVRx and 39.2% for the Sham VR. Cohen's *d* for the EaseVRx was 1.18, with combined results evidencing a large effect size and substantial clinical importance. Seventy-six percent (n=64) of EaseVRx participants achieved ≥30% reduction in Pain-Related Interference with Stress, and 68% (n=57) achieved ≥50% reduction. The VR Sham group effect size was 0.84, with combined results showing a large effect size and moderate clinical importance."

18) Results of any other analyses performed, including subgroup analyses and adjusted analyses, distinguishing pre-specified from exploratory

Does your paper address CONSORT subitem 18? *

All the below were prespecified.

"Global Impression of Change (PGIC). Participants in the EaseVRx group reported greater PGIC than Sham VR participants (4.13 vs. 3.11; p = 0.002).

While both groups evidenced improvement in pain coping symptoms, including pain catastrophizing, pain self-efficacy, and pain acceptance from pre-treatment to end of treatment, none of these improvements achieved statistical significance.

For PROMIS Physical Function, both treatment groups significantly improved throughout the study with the simple effect (i.e., effect of group within time slice) revealing superior functional improvement for the EaseVRx group relative to the Sham VR group (p=0.0224). Cohen's *ds* for the EaseVRx and VR Sham group were 0.64 and 0.35, respectively.

For PROMIS Sleep Disturbance, both treatment groups significantly improved over the course of the study with the simple effect (i.e., effect of group within time slice) revealing superior improvement for the EaseVRx group relative to the Sham VR group (p=0.0132). Cohen's *d* for the EaseVRx was 0.83 evidencing a large effect and substantial clinical importance.

Prescription Opioid and OTC Analgesic Use. Neither treatment group evidenced a significant change in MME dose from baseline to end of treatment. For OTC analgesic medication use, a

substantial decrease was observed in the EaseVRx group. While n=61 reported using OTC analgesics at baseline, n=50 reported use at post-treatment day 56 (p=0.01). For Sham VR, n=55 and n=56 reported OTC analgesic use at baseline and post-treatment, respectively (n/s).

Treatment Satisfaction, Likelihood to Recommend, and Likelihood to Continue Use. For the four summed satisfaction items, the EaseVRx group reported greater satisfaction with treatment compared to the Sham VR group (4.32 vs. 3.46 respectively; p<.001) Similarly, the EaseVRx group reported greater likelihood to recommend VR to someone else compared to the Sham VR group (8.72 vs 6.55, respectively; p<.001). Finally, EaseVRx participants reported greater likelihood to continue using VR if they could keep their headset compared to Sham VR (9.18 vs 7.23, respectively; p<.001).

VR Usability Ratings. Both treatment groups reported high usability with no statistical difference between groups (EaseVRx = 84.33; Sham VR = 81.16)."

18-i) Subgroup analysis of comparing only users

A subgroup analysis of comparing only users is not uncommon in ehealth trials, but if done, it must be stressed that this is a self-selected sample and no longer an unbiased sample from a randomized trial (see 16-iii).

subitem not at all important

1

2

3

4

5

essential

Does your paper address subitem 18-i?

Copy and paste relevant sections from the manuscript (include quotes in quotation marks "like this" to indicate direct quotes from your manuscript), or elaborate on this item by providing additional information not in the ms, or briefly explain why the item is not applicable/relevant for your study

Subgroup analysis was not part of this publication.

19) All important harms or unintended effects in each group

(for specific guidance see CONSORT for harms)

Does your paper address CONSORT subitem 19? *

Copy and paste relevant sections from the manuscript (include quotes in quotation marks "like this" to indicate direct quotes from your manuscript), or elaborate on this item by providing additional information not in the ms, or briefly explain why the item is not applicable/relevant for your study

“Data on cybersickness were collected at one month post-treatment and this lag introduces potential for recall bias, despite others documenting that participants readily recall cybersickness due to its specificity and salience.[17] Overall attrition was low (n=11) and it is possible that the two EaseVRx participants who left the study after receiving their headset did so due to cybersickness. No participants contacted study staff to report adverse events of any type.”

“Participants were provided with study staff contact information and encouraged to contact as needed and in the event of any problems using their device or with their treatment. Similar to other studies, cybersickness was assessed at the end of treatment.[17] However, due to a problem with the electronic survey, these data were not captured and the survey was readministered at one month post-treatment.’’

19-i) Include privacy breaches, technical problems

Include privacy breaches, technical problems. This does not only include physical “harm” to participants, but also incidents such as perceived or real privacy breaches [1], technical problems, and other unexpected/unintended incidents. “Unintended effects” also includes unintended positive effects [2].

subitem not at all important

1

2

3

4

5

essential

Does your paper address subitem 19-i?

Copy and paste relevant sections from the manuscript (include quotes in quotation marks "like this" to indicate direct quotes from your manuscript), or elaborate on this item by providing additional information not in the ms, or briefly explain why the item is not applicable/relevant for your study

No privacy issue were made during the study.

19-ii) Include qualitative feedback from participants or observations from staff/researchers

Include qualitative feedback from participants or observations from staff/researchers, if available, on strengths and shortcomings of the application, especially if they point to unintended/unexpected effects or uses. This includes (if available) reasons for why people did or did not use the application as intended by the developers.

subitem not at all important

1

2

3

4

5

essential

Does your paper address subitem 19-ii?

Copy and paste relevant sections from the manuscript (include quotes in quotation marks "like this" to indicate direct quotes from your manuscript), or elaborate on this item by providing additional information not in the ms, or briefly explain why the item is not applicable/relevant for your study

This data was not planned for this manuscript.

DISCUSSION

22) Interpretation consistent with results, balancing benefits and harms, and considering other relevant evidence

NPT: In addition, take into account the choice of the comparator, lack of or partial blinding, and unequal expertise of care providers or centers in each group

22-i) Restate study questions and summarize the answers suggested by the data, starting with primary outcomes and process outcomes (use)

Restate study questions and summarize the answers suggested by the data, starting with primary outcomes and process outcomes (use).

subitem not at all important

1

2

3

4

5

essential

Does your paper address subitem 22-i? *

Copy and paste relevant sections from the manuscript (include quotes in quotation marks "like this" to indicate direct quotes from your manuscript), or elaborate on this item by providing additional information not in the ms, or briefly explain why the item is not applicable/relevant for your study

“We conducted the first placebo-controlled RCT of home-based therapeutic VR in a national sample of individuals with CLBP. We hypothesized that an 8-week pain relief skills VR program (EaseVRx) would be superior to Sham VR at post-treatment (day 56) for our primary outcomes: average pain intensity and pain-related interference with activity, mood, sleep and stress. While both study groups had significant reductions in pain and all domains of pain-related interference, EaseVRx evidenced superior treatment effects for all primary outcomes except sleep interference.”

22-ii) Highlight unanswered new questions, suggest future research

Highlight unanswered new questions, suggest future research.

subitem not at all important

1

2

3

4

5

essential

Does your paper address subitem 22-ii?

Copy and paste relevant sections from the manuscript (include quotes in quotation marks "like this" to indicate direct quotes from your manuscript), or elaborate on this item by providing additional information not in the ms, or briefly explain why the item is not applicable/relevant for your study

“Future VR research should examine medication use in greater detail and with higher frequency data capture.”

“Future research may better capture potential VR adverse effects by assessing these factors in the first week of treatment. Interpretation of data on prescription opioid use was limited by low frequency sampling methods that are subject to recall bias and poor data accuracy. Opioid prescriptions often allow ‘as needed’ flexibilities in medication use and future research designs may benefit from high frequency sampling methods which improve data accuracy.”

20) Trial limitations, addressing sources of potential bias, imprecision, and, if relevant, multiplicity of analyses

20-i) Typical limitations in ehealth trials

Typical limitations in ehealth trials: Participants in ehealth trials are rarely blinded. Ehealth trials often look at a multiplicity of outcomes, increasing risk for a Type I error. Discuss biases due to non-use of the intervention/usability issues, biases through informed consent procedures, unexpected events.

subitem not at all important

1

2

3

4

5

essential

Does your paper address subitem 20-i? *

Copy and paste relevant sections from the manuscript (include quotes in quotation marks "like this" to indicate direct quotes from your manuscript), or elaborate on this item by providing additional information not in the ms, or briefly explain why the item is not applicable/relevant for your study

“Several limitations bear consideration when evaluating the study results. With the exception of device use metrics, all data were self-reported. The study was untethered from medical care and thus there was no ability to confirm pain diagnoses or analgesic prescription information. The study sample was predominantly female, white, college educated, and Internet savvy; as such, findings may not generalize to individuals with disparate demographic characteristics. These findings are consistent with previous evidence showing that highly educated females are more likely to use self-care mobile health technologies, particularly those with mindfulness-based content (Bol et al., 2018). As e-health literacy and awareness increases in clinicians and the general population, it is likely that health technologies (including therapeutic VR) will benefit other demographics (Neter and Brainin 2012). Additionally, the study was conducted in individuals with CLBP and findings may not generalize to other chronic pain conditions.

Data on cybersickness were collected at one month post-treatment and this lag introduces potential for recall bias, despite others documenting that participants readily recall cybersickness due to its specificity and salience.[17] Overall attrition was low (n=11) and it is possible that the two EaseVRx participants who left the study after receiving their headset did so due to cybersickness. No participants contacted study staff to report adverse events of any type. Data on sex differences for cybersickness are mixed, with some reporting a female preponderance (Golding 2006) while a recent meta-analysis suggests no sex effect (Dimitrios 2020). Within the context of our limitations, we highlight low reports of cybersickness in a predominantly female sample.”

21) Generalisability (external validity, applicability) of the trial findings

NPT: External validity of the trial findings according to the intervention, comparators, patients, and care providers or centers involved in the trial

21-i) Generalizability to other populations

Generalizability to other populations: In particular, discuss generalizability to a general Internet population, outside of a RCT setting, and general patient population, including applicability of the study results for other organizations

subitem not at all important

1

2

3

4

5

essential

Does your paper address subitem 21-i?

Copy and paste relevant sections from the manuscript (include quotes in quotation marks "like this" to indicate direct quotes from your manuscript), or elaborate on this item by providing additional information not in the ms, or briefly explain why the item is not applicable/relevant for your study

“The study sample was predominantly female, white, college educated, and Internet savvy; as such, findings may not generalize to individuals with disparate demographic characteristics.”

“Additionally, the study was conducted in individuals with CLBP and findings may not generalize to other chronic pain conditions.”

21-ii) Discuss if there were elements in the RCT that would be different in a routine application setting

Discuss if there were elements in the RCT that would be different in a routine application setting (e.g., prompts/reminders, more human involvement, training sessions or other co-interventions) and what impact the omission of these elements could have on use, adoption, or outcomes if the intervention is applied outside of a RCT setting.

subitem not at all important

1

2

3

4

5

essential

Does your paper address subitem 21-ii?

Copy and paste relevant sections from the manuscript (include quotes in quotation marks "like this" to indicate direct quotes from your manuscript), or elaborate on this item by providing additional information not in the ms, or briefly explain why the item is not applicable/relevant for your study

"The study was untethered from medical care and thus there was no ability to confirm pain diagnoses or analgesic prescription information."

OTHER INFORMATION

23) Registration number and name of trial registry

Does your paper address CONSORT subitem 23? *

Copy and paste relevant sections from the manuscript (include quotes in quotation marks "like this" to indicate direct quotes from your manuscript), or elaborate on this item by providing additional information not in the ms, or briefly explain why the item is not applicable/relevant for your study

Trial registration: ClinicalTrials.gov, NCT04415177
https://clinicaltrials.gov/ct2/show/NCT04415177

24) Where the full trial protocol can be accessed, if available

Does your paper address CONSORT subitem 24? *

Cite a Multimedia Appendix, other reference, or copy and paste relevant sections from the manuscript (include quotes in quotation marks "like this" to indicate direct quotes from your manuscript), or elaborate on this item by providing additional information not in the ms, or briefly explain why the item is not applicable/relevant for your study

The full trial protocol can be accessed on JMIR protocol journal.

25) Sources of funding and other support (such as supply of drugs), role of funders

Does your paper address CONSORT subitem 25? *

Copy and paste relevant sections from the manuscript (include quotes in quotation marks "like this" to indicate direct quotes from your manuscript), or elaborate on this item by providing additional information not in the ms, or briefly explain why the item is not applicable/relevant for your study

Funding

AppliedVR, Inc supported this study.

X27) Conflicts of Interest (not a CONSORT item)

X27-i) State the relation of the study team towards the system being evaluated

In addition to the usual declaration of interests (financial or otherwise), also state the relation of the study team towards the system being evaluated, i.e., state if the authors/evaluators are distinct from or identical with the developers/sponsors of the intervention.

subitem not at all important

1

2

3

4

5

essential

Does your paper address subitem X27-i?

Copy and paste relevant sections from the manuscript (include quotes in quotation marks "like this" to indicate direct quotes from your manuscript), or elaborate on this item by providing additional information not in the ms, or briefly explain why the item is not applicable/relevant for your study

“Disclosures and Conflicts of Interest

Drs. Garcia and Maddox and Ian Mackey are employees of AppliedVR, Inc. Joshua Sackman is president of AppliedVR, Inc. Dr. Darnall is chief science advisor for AppliedVR, Inc. Drs. Birckhead, Krishnamurthy, and Salmasi are consultants for AppliedVR, Inc.

About the CONSORT EHEALTH checklist”

As a result of using this checklist, did you make changes in your manuscript? *

yes, major changes

yes, minor changes

no

What were the most important changes you made as a result of using this checklist?

We luckily had all the required elements requested within the manuscript.

How much time did you spend on going through the checklist INCLUDING making changes in your manuscript *

6 hours

As a result of using this checklist, do you think your manuscript has improved? *

yes

no

Other:

Would you like to become involved in the CONSORT EHEALTH group?

This would involve for example becoming involved in participating in a workshop and writing an "Explanation and Elaboration" document

yes

no

Other:

Any other comments or questions on CONSORT EHEALTH

Your answer

STOP - Save this form as PDF before you click submit

To generate a record that you filled in this form, we recommend to generate a PDF of this page (on a Mac, simply select "print" and then select "print as PDF") before you submit it.

When you submit your (revised) paper to JMIR, please upload the PDF as supplementary file.

Don't worry if some text in the textboxes is cut off, as we still have the complete information in our database. Thank you!

Final step: Click submit !
